# *C*-Methylation of *S*-adenosyl-L-Methionine Occurs Prior to Cyclopropanation in the Biosynthesis of 1-Amino-2-Methylcyclopropanecarboxylic Acid (Norcoronamic Acid) in a Bacterium

**DOI:** 10.3390/biom10050775

**Published:** 2020-05-16

**Authors:** Chitose Maruyama, Yukiko Chinone, Shusuke Sato, Fumitaka Kudo, Kosuke Ohsawa, Junya Kubota, Junko Hashimoto, Ikuko Kozone, Takayuki Doi, Kazuo Shin-ya, Tadashi Eguchi, Yoshimitsu Hamano

**Affiliations:** 1 Department of Bioscience, Fukui Prefectural University, 4-1-1 Yoshida-Gun, Fukui 910-1195, Japan; c-maruyama@fpu.ac.jp (C.M.); s1873013@g.fpu.ac.jp (Y.C.); 2 Department of Chemistry, Tokyo Institute of Technology, 2-12-1 O-okayama, Meguro-ku, Tokyo 152-8551, Japan; satou.s.ae@m.titech.ac.jp (S.S.); fkudo@chem.titech.ac.jp (F.K.); eguchi@chem.titech.ac.jp (T.E.); 3 Graduate School of Life Sciences, Tohoku University, 6-3 Aza-aoba, Aramaki, Aoba-ku, Sendai 980-8578, Japan; kosuke@mail.pharm.tohoku.ac.jp (K.O.); junya.kubota.s7@gmail.com (J.K.); doi_taka@mail.pharm.tohoku.ac.jp (T.D.); 4 Japan Biological Informatics Consortium (JBIC), 2-4-7 Aomi, Koto-ku, Tokyo 135-0064, Japan; junko.hashimoto@aist.go.jp (J.H.); ikuko-kozone@aist.go.jp (I.K.); 5 National Institute of Advanced Industrial Science and Technology, 2-4-7 Aomi, Koto-ku, Tokyo 135-0064, Japan; k-shinya@aist.go.jp; 6 The Biotechnology Research Center, The University of Tokyo, 1-1-1 Yayoi, Bunkyo-ku, Tokyo 113-8657, Japan; 7 Collaborative Research Institute for Innovative Microbiology, The University of Tokyo, 1-1-1 Yayoi, Bunkyo-ku, Tokyo 113-8657, Japan

**Keywords:** 1-amino-2-methylcyclopropanecarboxylic acid, 1-aminocyclopropanecarboxylic acid (ACC), ACC synthase, radical *S*-adenosyl-L-methionine (SAM) methyltransferase

## Abstract

Many pharmacologically important peptides are bacterial or fungal in origin and contain nonproteinogenic amino acid (NPA) building blocks. Recently, it was reported that, in bacteria, a cyclopropane-containing NPA 1-aminocyclopropanecarboxylic acid (ACC) is produced from the L-methionine moiety of *S*-adenosyl-L-methionine (SAM) by non-canonical ACC-forming enzymes. On the other hand, it has been suggested that a monomethylated ACC analogue, 2-methyl-ACC (MeACC), is derived from L-valine. Therefore, we have investigated the MeACC biosynthesis by identifying a gene cluster containing bacterial MeACC synthase genes. In this gene cluster, we identified two genes, *orf29* and *orf30*, which encode a cobalamin (B12)-dependent radical SAM methyltransferase and a bacterial ACC synthase, respectively, and were found to be involved in the MeACC biosynthesis. In vitro analysis using their recombinant enzymes (rOrf29 and rOrf30) further revealed that the ACC structure of MeACC was derived from the L-methionine moiety of SAM, rather than L-valine. In addition, rOrf29 was found to catalyze the *C*-methylation of the L-methionine moiety of SAM. The resulting methylated derivative of SAM was then converted into MeACC by rOrf30. Thus, we demonstrate that *C*-methylation of SAM occurs prior to cyclopropanation in the biosynthesis of a bacterial MeACC (norcoronamic acid).

## 1. Introduction

Many pharmacologically important peptides are bacterial or fungal in origin and are nonribosomally synthesized by multimodular enzymes, referred to as nonribosomal peptide synthetases (NRPSs) [1,2,3]. Using assembly-line logic, comprising multiple modules, NRPSs utilize a thiotemplated mechanism to activate, tether, and modify amino-acid building blocks, sequentially elongating the peptide chain before releasing the complete peptide [1,2,3]. In general, the order and number of modules of an NRPS system determine the sequence and length of the peptide product. In this machinery, an adenylation (A) domain in the module plays an important role in selecting and activating amino-acid building blocks as aminoacyl adenylates with ATP. Therefore, the substrate specificities of A domains determine the amino-acid components of nonribosomal peptides. Interestingly, nonribosomal peptides often contain nonproteinogenic amino acids (NPAs) in their chemical structures [4], since the A domain can accept not only the standard amino acids but also NPAs as building blocks [5]. This advantageous feature of NPA incorporation makes a substantial contribution to the structural diversity of peptide natural products, resulting in the potent biological activities of these compounds. Moreover, the NPA-containing peptides are more biologically stable due to their resistance to amide hydrolases, such as peptidase and protease. Consequently, screening of cryptic NPA-containing peptides has become more important for effective drug discoveries. This strategy is also supported by the fact that therapeutically important nonribosomal peptides, such as vancomycin, daptomycin, and cyclosporin, are NPA-containing peptides.

**Figure 1:**
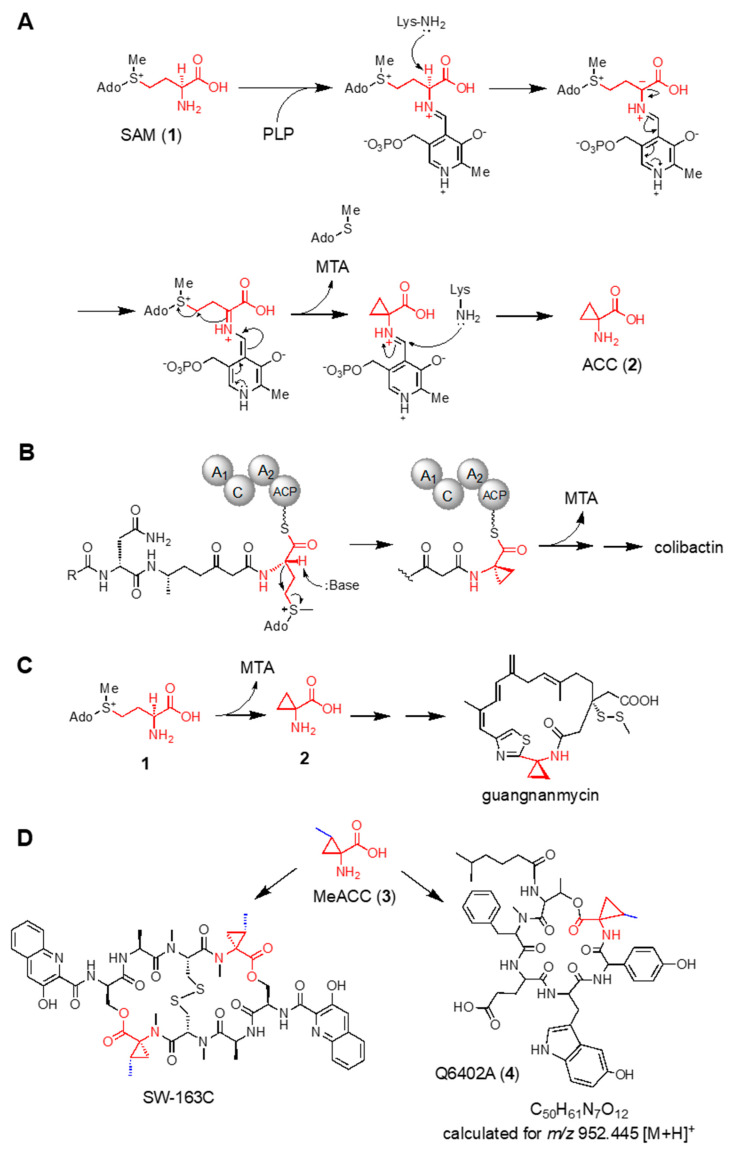
Chemical structures. (**A**) 1-aminocyclopropanecarboxylic acid (ACC) formation catalyzed by plant ACC synthases. (**B**) Formation of the ACC building block in the colibactin biosynthesis. (**C**) ACC formation catalyzed by GmnY in the guangnanmycin biosynthesis. (**D**) Peptide natural products with the 2-methyl-ACC (MeACC) building block.

Knowledge about the biosynthetic route to the major classes of NPAs, which are incorporated into nonribosomal peptides, has steadily increased over the last two decades [4,5,6]. In the present study, we focused on the biosynthesis of NPAs harboring a cyclopropane ring, as the inherent ring strain present in the small ring moiety is frequently responsible for the biological activities of these compounds [7,8]. The simplest cyclopropane-containing NPA is 1-aminocyclopropanecarboxylic acid (ACC) (2), which has been isolated from many fruits and plant tissues (Figure 1A). Compound 2 is known to be the crucial and immediate precursor of the important plant hormone ethylene, which is involved in senescence, fruit ripening, and interspecies communication in plants [8]. Furthermore, in 1979, the plant ACC scaffold was found to be derived from *S*-adenosyl-L-methionine (SAM) (1) (Figure 1A). The cyclopropanation with the production of 5′-methylthioadenosine (MTA) is mediated by pyridoxal-5′-phosphate (PLP)-dependent aminotransferases (ACC synthases) [9]. However, very recently, it was reported that microorganisms employ alternative machineries for the ACC formation to produce ACC-containing secondary metabolites. In the colibactin biosynthesis, the ACC moiety was derived from 1, but the cyclopropanation was catalyzed not by the PLP-dependent aminotransferase, but rather by the synergic action of NRPS and polyketide synthetase (Figure 1B) [10]. The guangnanmycin biosynthesis employs a novel bacterial ACC synthase, GnmY, to form 2 and MTA from 1 using a PLP-dependent mechanism similar to that of the plant ACC synthases (Figure 1C) [11]. However, surprisingly, GnmY does not share homology with any ACC synthases from plants, although its primary structure is classified as a PLP-dependent aminotransferase.

On the other hand, the biosynthesis of the next-simplest cyclopropane-containing NPA, 1-amino-2-methylcyclopropanecarboxylic acid (MeACC) (3), remains unclear (Figure 1D). Compound 3 (termed norcoronamic acid) was found in SW-163C [12,13] and its analogues. The formation of 3 in their biosynthesis is still speculative; a radical SAM protein (Swb7) and a PLP-dependent aminotransferase (Swb6) could work together closely to form 3 from L-valine via radical cyclopropanation in the SW-163 biosynthesis [14]. However, an alternative biosynthetic route to 3 has been suggested by the fact that the amino-acid sequence of Swb6 is homologous with that of GnmY, utilizing 1 as the substrate.

Here, we report that the biosynthesis of the MeACC building block is mediated by two unique enzymes, Orf29 and Orf30, which are the Swb7 and Swb6/GnmY homologues, respectively; Orf29 (a radical SAM methyltransferase) catalyzes the *C*-methylation of 1, and the resulting compound is catalytically transformed to 3 by Orf30 (PLP-dependent aminotransferase) in vitro. This finding expands our knowledge of cyclopropane-containing NPA biosynthesis.

## 2. Materials and Methods

### 2.1. Chemicals

All chemicals (1-aminocyclopropanecarboxylic acid (ACC), *S*-adenosyl-L-methionine (SAM), *S*-adenosyl-L-homocysteine (SAH), [1-^13^C]-L-methionine, and [5-^13^C]-L-methionine) were purchased from Tokyo Chemical Industry (Tokyo, Japan), Sigma-Aldrich Japan Inc. (Tokyo, Japan), and Cambridge Isotope Laboratories (CIL; Tewksbury, MA, USA). The authentic standard compounds of (*1S,2R*)-MeACC and (*1R,2R*)-MeACC were prepared according to the procedures reported previously [15,16]. Oligonucleotides were obtained from Eurofines Genomics (Tokyo, Japan). All other chemicals used were of analytical grade.

### 2.2. Bacterial Strains, Plasmids, and Culture Media

Bacterial strains and plasmids used in this study are summarized in Table S1.

2× SK No.2 medium, consisting of 4% (*w*/*v*) soluble starch, 1% (*w/v*) glucose, 1% (*w*/*v*) yeast extract (Difco Laboratories, Franklin Lakes, NJ, USA), 0.6% (*w/v*) beef extract (Difco), 0.6% (*w*/*v*) peptone, 0.04% (*w*/*v*) KH_2_PO_4_, and 0.12% (*w*/*v*) MgSO_4_ and 7H_2_O (pH7.6) was used for the heterologous expression experiments, using *Streptomyces lividans* TK23 as a host strain. The *S. lividans* TK23 strain was also grown in S10.3 medium, consisting of 10.3% (*w*/*v*) sucrose, 3% (*w*/*v*) glucose, 1.5% (*w*/*v*) soytone (Difco), 0.1% (*w*/*v*) glycine, 2.7 mM CaCl_2_, and 5 mM MgCl_2_ (pH 7.2). *Escherichia coli* EcSUF derivatives were cultured in Terrific Broth (TB) medium, containing 1% (*w*/*v*) glycerol, 2.4% (*w*/*v*) yeast extract (Difco), 1.2% (*w*/*v*) tryptone (Difco), 0.94% (*w*/*v*) K_2_HPO_4_, 0.22% (*w*/*v*) KH_2_PO_4_, 0.01% (*w*/*v*) ammonium ferric citrate, and 7.845% (*w*/*v*) FeSO_4_(NH_4_)_2_SO_4_·6H_2_O.

### 2.3. Cloning of the Biosynthetic Gene Cluster With Genes Homologous to Swb7 and Swb6/gnmY

**Figure 2:**
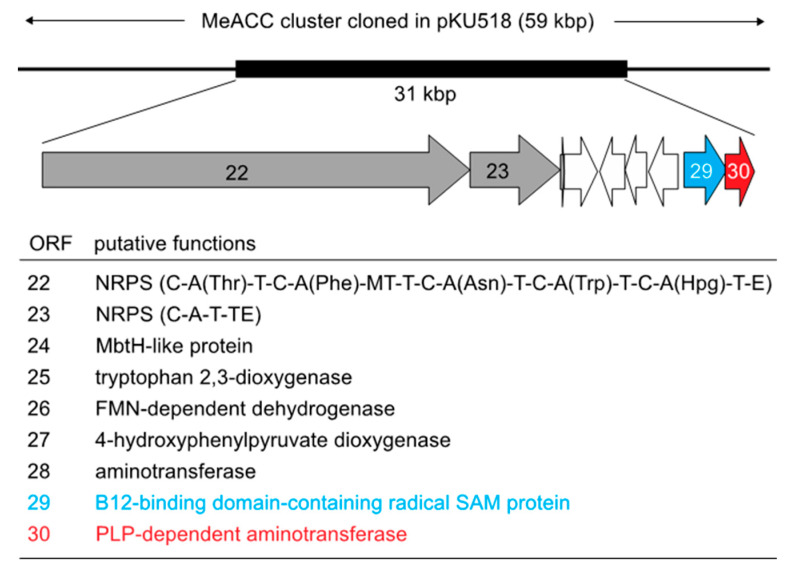
Gene organization of the MeACC cluster. The 59k bp DNA fragment was deposited in the DNA Database of Japan (DDBJ), European Molecular Biology Laboratory (EMBL), and GenBank databank under accession number LC535008. Condensation domain (C); thiolation domain (T; peptide carrier protein domain); methyltransferase domain (MT); epimerase domain (E), and thioesterase domain (TE).

In the draft genome database of our laboratory stock strains, antiSMASH analysis [17] showed that the genome DNA of *Streptomyces violaceusniger* 4521-SVS3 has the gene cluster carrying two genes, *orf29* (accession number, BCD33697) and *orf30* (BCD33698), which are homologous to *swb7* and *swb6/gnmY*, respectively (Figure 2). In addition, two NRPS genes (*orf22* and *orf23*) were found within the gene cluster, suggesting that this gene cluster is responsible for the production of a nonribosomal peptide, containing the MeACC building block. We therefore designated the gene cluster as the MeACC cluster. After the construction of the genome library of the 4521-SVS3 strain using the BAC vector pKU518, according to a previously reported method [18], a BAC clone containing the entire MeACC cluster was screened by PCR amplification using two sets of primers (orf21-F and orf21-R; orf30-F and orf30-R, Table S2). The positive clone (pKU518_MeACC) carrying the whole MeACC cluster was selected, and the insert sequence was confirmed by end-sequencing (Table S1 and Figure 2). The 59 kbp DNA fragment was deposited in the DNA Database of Japan (DDBJ), European Molecular Biology Laboratory (EMBL), and GenBank databank under accession number LC535008.

### 2.4. Expression of the MeACC Cluster in a Heterologous Host Strain, S. Lividans TK23

The BAC clones, pKU518_MeACC and pKU518 (Table S1), were respectively introduced into *S. lividans* TK23 by standard procedures [19]. The two resulting transformants, TK23_MeACC and TK23_empty (Table S1), harboring pKU518_MeACC and pKU518, respectively, were cultured in 2×SK No.2 medium for 6 days at 28 °C. In the feeding experiments, using ^13^C-labeled L-methionine, 2×SK No.2 medium supplemented with 0.1% [1-^13^C]-L-methionine or 0.1% [5-^13^C]-L-methionine was employed. To terminate cultivation and extract the peptide compounds, an equivalent volume of acetone was added, and the culture broths were shaken for 9 h at 15 °C. After centrifugation, the resulting supernatants were evaporated to remove the acetone and were then analyzed by high-performance liquid chromatography and high-resolution electrospray ionization mass spectrometry (HPLC-HR-ESI-MS) analysis (maXis plus; Bruker) using a reversed-phase column (Sunshell RP-AQUA, 2.6 μm, 50 × 2.1 mm; ChromaNik Technologies, Osaka, Japan) at 40 °C at a flow rate of 0.3 mL/min and with a linear gradient of acetonitrile in water in 0.1% (*v/v*) formic acid run over 16 min (5–100% (*v/v*) acetonitrile for 16 min).

### 2.5. Inactivation of the orf29 and orf30 Genes

To investigate the function of *orf29* or *orf30*, we constructed BAC clones carrying an MeACC cluster, in which the *orf29* or *orf30* gene was inactivated by an in-frame deletion with a PCR-targeted mutagenesis strategy [20]. The resulting BAC vectors, pKU518_MeACC_*∆orf29* and pKU518_MeACC_*∆orf30* (Table S1), were respectively introduced into *S. lividans* TK23, and their transformants (TK23_MeACC_*∆orf29* and TK23_MeACC_*∆orf30*) (Table S1) were cultured in 2×SK No.2 medium with or without 0.2% (*w*/*v*) ACC for 6 days at 28 °C. To confirm the productivity of the peptide compounds from the inactivated gene clusters, the culture broths were analyzed by HPLC-HR-ESI-MS, as described above (Section 2.4).

### 2.6. Overexpression and Purification of the orf30 Recombinant Enzyme

The following two PCR primers were designed and used to amplify the *orf30* gene: pHSA81_*orf30*-F and pHSA81_C8His-*orf30*-R (Table S2). The PCR product was ligated with the expression vector, pHSA81 (a gift from Dr. Kobayashi, University of Tsukuba, Tsukuba, Japan). After confirmation of the DNA sequence, the resulting plasmid (pHSA81_*orf30*_C8His, Table S1) was introduced into *S. lividans* TK23 for expression as a *C*-terminally 8×His-tagged fusion protein by a standard procedure [19]. The transformant (TK23_rOrf30/C8His; Table S1) was inoculated into S10.3 medium, containing 20 μg/ml thiostrepton. After growth for 3 days at 28 °C, cells were harvested from 50 mL culture broth by centrifugation at 6000× *g* for 15 min, resuspended in 5 mL Buffer A (50 mm sodium phosphate buffer (NaPB), 10% glycerol, 300 mM NaCl, 0.1 mM PLP, and pH 8.0), containing 10 mM imidazole and sonicated on ice. Insoluble material was removed by centrifugation at 12,000× *g* for 15 min. The supernatant was run on a 1 mL nickel-nitriloacetic acid (Ni-NTA) Sepharose column (Qiagen) that had been pre-equilibrated with 5 mL Buffer A, containing 10 mM imidazole. The column was washed with 5 mL Buffer A, containing 20 mM imidazole, and recombinant enzymes were eluted with 1 mL Buffer A, containing 250 mM imidazole and used for in vitro enzyme reactions.

The molecular weight of the purified protein (rOrf30) was determined by SDS-PAGE and gel-exclusion chromatography, using a SunSec diol-30 column (ChromaNik Technologies).

### 2.7. In Vitro Enzyme Reactions with rOrf30

A reaction mixture (100 μL) consisting of 50 mM NaPB (pH 8.0), 500 μM SAM or L-methionine, and 100 μg/mL rOrf30 was incubated at 30 °C for 15 h. The enzyme reaction was quenched by heating at 100 °C for 1 min and the denatured enzyme was removed by centrifugation. The reaction product was derivatized with 3-aminopyridinyl-*N*-hydroxysuccinimidyl carbamate (APDS), according to the manufacturer’s instructions (FUJIFILM Wako Pure Chemical, Japan), and was then analyzed by HPLC-HR-ESI-MS using a reversed-phase column (Sunniest RP-AQUA, 3 μm, 150 × 2.1 mm; ChromaNik Technologies) at 40 °C at a flow rate of 0.3 mL/min and with a two-step linear gradient of acetonitrile in water in 0.1% (*v/v*) heptafluorobutyric acid (HFBA) (Wako, Japan) run over 18 min (2% (*v/v*) acetonitrile for 5 min, 2–50% (*v/v*) acetonitrile for 11 min, and 50–98% (*v/v*) acetonitrile for 2 min).

Kinetic assays were performed under conditions identical to those described above, except that the reaction time (60 min) was reduced to enable the measurement of steady-state kinetic parameters. All assays were carried out under linear conditions. The *K*_m_ and *K*_cat_ values were calculated from curves fitting to the Michaelis–Menten equation using GraphPad Prism8 software. The kinetic analysis was performed in triplicate.

### 2.8. Construction of E. Coli Strains Expressing the Suf and/or Btu Operons

A plasmid pRKSUF017 [21] carrying the suf operon (a gift from Dr. Takahashi, Saitama University, Saitama, Japan) was introduced to *E. coli* C41 (DE3) for the (4Fe-4S) cluster reconstitution. The resulting strain, EcSuf (Table S1), was used as a host strain for the heterologous co-expression experiment using two genes, orf29 and orf30 (see Section 2.9). In addition, we constructed a plasmid, pBAD24_BtuCEDFB, which carries the cobalamin uptake genes, according to the method described by Booker et al. [22]. The synthetic DNA fragments (Table S3) containing five genes, btuC, btuE, btuD, btuF, and btuB, which were designed according to the plasmid map of pBAD42-BtuCEDFB, were obtained from Eurofins Genomics (Tokyo, Japan). The fragment 1 digested with NcoI and PvuI was inserted into the same restriction enzyme sites of a pRSFDuet-1 vector to obtain the plasmid pRSF_btuCE’. The fragment 2 was digested with PvuI and KpnI and then ligated into the pRSF_btuCE’ construct and digested with the same enzymes to get the plasmid pRSF_btuCEDFB’. The fragment 3 was digested with HindIII and XhoI and then ligated into the pRSF_btuCEDFB’ construct and digested with the same enzymes to get the plasmid pRSF_btuCEDFB. Finally, the NcoI–XbaI fragment of the plasmid pRSF_btuCEDFB was inserted into the same restriction enzyme sites of a pBAD24 vector [23] (purchased from the Yale Coli Genetic Stock Center) to construct the plasmid pBAD24_BtuCEDFB.

The plasmids, pBAD24_BtuCEDFB and pRKSUF017, were introduced into *E. coli* BL21(DE3). The resulting strain, EcSufBtu (Table S1), was employed for the overexpression of rOrf29 (see Section 2.10).

### 2.9. Heterologous Co-Expression of Two Genes, orf29 and orf30, in E. coli

To amplify the *orf29* and *orf30* genes, the following two sets of PCR primers were used: pETDuet-1*_orf29-*F/pETDuet-1*_orf29-*R and pETDuet-1*_orf30-*F/pETDuet-1*_orf30-*R (Table S2). Both PCR products were ligated with a pETDuet-1 vector to yield the expression vector pETDuet-1_*orf29*_*orf30* (Table S1). In addition, pETDuet-1_*orf29* was also constructed for a control experiment. These two constructed vectors and pETDuet-1(empty) were respectively introduced into the EcSuf strain, which expressed the *suf* operon for iron-sulfur cluster reconstitution (Table S1). The resulting transformants, EcSuf_*orf29*_*orf30*, EcSuf_*orf29*, and EcSuf_empty (Table S1), were cultured in TB medium, supplemented with 200 μM L-cysteine, 20 μM methylcobalamine, 0.1% (*w*/*v*) L-methionine, and 1 mM isopropyl-β-D-thiogalactopyranoside (IPTG) for 42 h at 28 °C. Additionally, the EcSuf_*orf29* strain was grown in TB medium, supplemented with 0.2% (*w*/*v*) ACC (2). To terminate the cultivation and extract MeACC (3), an equivalent volume of acetone was added, and the culture broths were shaken for 9 h at 15 °C. After centrifugation, the resulting supernatants were evaporated to remove the acetone. Compound 3 in the extract was derivatized with APDS and was then analyzed by HPLC-HR-ESI-MS under the same conditions employed for the ACC analysis (Section 2.7). (*1R,2R*)-MeACC (5) and (*1S,2R*)-MeACC (6) were used as authentic standards.

### 2.10. In Vitro Enzyme Reactions with rOrf29

The following two PCR primers were designed and used to amplify the *orf29* gene: pET28_*orf29*-F and pET28_*orf29*-R (Table S2). The PCR product was ligated with the expression vector pET28. After confirmation of the DNA sequence, the resulting plasmid (pET28_*orf29*; Table S1) was introduced into the *E. coli* EcSufBtu strain (see Section 2.8) (Table S1), which expresses the *suf* and *btu* operons. The resulting transformant, EcSufBtu_orf29, was inoculated into LB medium, containing 50 μg/mL kanamycin, 100 μg/mL ampicillin, and 5 μg/mL tetracycline. After growth overnight at 37 °C, the culture (1 mL) was inoculated into 200 mL of M9-ethanolamine medium [24], containing 50 μg/mL kanamycin, 100 μg/mL ampicillin, and 5 μg/mL tetracycline, and incubated at 37 °C with 200 rpm agitation until the OD600 = 0.2. L-Arabinose was added to a final concentration of 1 mg/mL, followed by re-cultivation until the OD600 = 0.6. The culture was cooled down on ice, and then FeSO_4_(NH_4_)_2_SO_4_ and L-cysteine were added to each final concentration of 0.2 mM each. Protein expression was induced by the addition of IPTG to a final concentration of 0.2 mM. Cultivation was continued at 15 °C for 20–24 h with 80 rpm agitation. The cells were harvested by centrifugation, washed with buffer B (50 mM HEPES-Na, 10% glycerol, 300 mM NaCl, and pH 8.0), and stored at −30 °C until use. The wet cells were transferred into a glovebox and subsequent disruption and purification were conducted under anaerobic conditions ([O_2_] ≤ 5 ppm). The wet cells (3 g) were suspended in degassed buffer B (30 mL). The suspension of cells was disrupted by sonication, with sonication bursts of 5 s, with a 5 s interval (total 4 min) on the cooled aluminum beads. Cell debris was removed by centrifugation (14,000× *g*, 20 min, at 4 °C). The supernatant was loaded onto a TALON resin (Clontech, Mountain View, CA) column that had been pre-equilibrated with buffer B. The column was washed with buffer B, containing 10 mM imidazole. Orf29 expressed as an *N*-terminally 6×His-tagged fusion protein (rOrf29) was eluted with buffer B, containing 200 mM imidazole. The protein solution was collected and desalted with a PD-10 desalting column (GE Healthcare, Buckinghamshire, UK).

To reconstitute the iron-sulfur cluster in rOrf29, the purified enzyme was incubated with 5 m dithiothreitol (DTT) for 15 min at room temperature and was then incubated with 10 equivalent mole of Na_2_S and 10 equivalent mole of FeSO_4_(NH_4_)_2_SO_4_ for 30 min at room temperature. After the confirmation of the reduced form of [4Fe-4S]^+^ by UV-VIS spectroscopic analysis, the reconstituted rOrf29 was used for enzyme assays in vitro. A reaction mixture consisting of 50 mM HEPES-Na (pH 8.0), 300 mM NaCl, 0.1 mM methylcobalamine, 1 mM methylviologen, 4 mM NADH, 10 mM DTT, 10% (*v*/*v*) glycerol, 1 mM SAM (1), and 12 μM rOrf29 was incubated at 28 °C for 16 h under an anaerobic condition. The reaction mixture was then analyzed by HPLC-HR-ESI-MS using a hydrophilic interaction chromatography (HILIC) column (TSK-gel Amide-80, 3 μm, 150 × 2.0 mm; TOSOH) at 40 °C at a flow rate of 0.2 mL/min and with a three step linear gradient of acetonitrile in water in 0.1% (*v/v*) formic acid and 10 mM ammonium formate run over 20 min (80% (*v/v*) acetonitrile for 5 min, 80–50% (*v/v*) acetonitrile for 10 min, and 50–10% (*v/v*) acetonitrile for 5 min). To confirm the production of 5′-deoxyadenosine (Ado-CH_3_) in the enzyme reaction, the reaction mixture was further analyzed by HPLC-ESI-MS using a reversed-phase column (TSK-gel ODS-100Z, 3 μm, 150 × 2.0 mm; TOSOH) at 40 °C at a flow rate of 0.2 mL/min and with a two-step linear gradient of acetonitrile in water run over 15 min (5% (*v/v*) acetonitrile for 5 min and 5–95% (*v/v*) acetonitrile for 10 min).

In addition, 0.5 μM rOrf30 or 0.5 mM ACC was added to the rOrf29 enzyme reaction to confirm the productivity of 3, and the absolute configuration of 3 was determined by the advanced Marfey’s method [25,26].

## 3. Results

### 3.1. Genome Mining of the Biosynthetic Gene Cluster With Genes Homologous to swb7 and swb6/gnmY

To explore a biosynthetic gene cluster involved in the biosynthesis of NPA-containing peptide compounds, we carried out a survey of the *Streptomyces* genomes in our lab stock strains. Using antiSMASH, we found a gene cluster that included two genes, *orf29* (accession number, BCD33697) and *orf30* (BCD33698), which are homologous to *swb7* and *swb6/gnmY*, respectively. The deduced amino acid sequence of the *orf29* gene product (Orf29) had a similarity with that of Swb7 (69% identity) (Figure S1 and Table S4), and Orf30 shared identity with Swb6 (69%) and GnmY (40%) (Figure S2 and Table S5).

The antiSMASH analysis further showed the possibility that two NRPS genes (*orf22* and *orf23*) in the flanking region of the *orf29* and *orf30* genes are involved in the biosynthesis of a hexa-peptide compound based on their domain architectures (Figure 2); *orf22* and *orf23* have five and one A domains, respectively, and the substrates of these six A domains were predicted to be L-threonine, L-phenylalanine, L-aspartate, L-tryptophan, hydroxy phenylglycine, and L-valine by NRPSpredictor2 [27] (Table S6). In addition, the *orf26*, *orf27,* and *orf28* genes were predicted to encode flavin mononucleotide (FMN)-dependent dehydrogenase, 4-hydroxyphenylpyruvate dioxygenase, and alanine-glyoxylate aminotransferase, respectively, which would participate in the biosynthesis of hydroxy phenylglycine [28]. From these analyses, we speculated that the gene cluster produced a MeACC-containing peptide. Therefore, we designated the gene cluster as the MeACC cluster.

### 3.2. Heterologous Expression of the MeACC Cluster in S. lividans TK23

**Figure 3:**
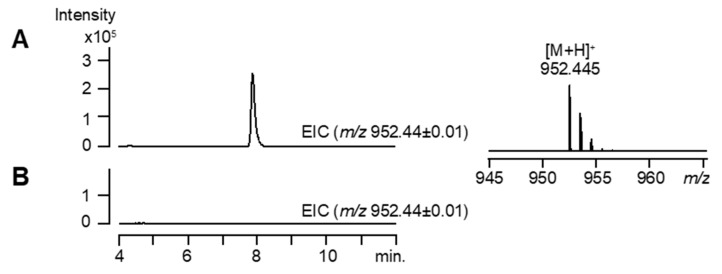
Heterologous expression of the MeACC cluster in *S. lividans* TK23. (**A**) The MeACC cluster was cloned (pKU518_MeACC) and introduced into a heterologous host strain, *S. lividans* TK23. The resulting transformant, TK23_MeACC, was cultured and analyzed by high-performance liquid chromatography and high-resolution electrospray ionization mass spectrometry (HPLC-HR-ESI-MS). The extracted ion chromatogram (EIC) for *m/z* 952.44 ± 0.01 is shown. (**B**) The TK23 strain harboring the pKU518 empty vector (TK23_empty) was cultured and analyzed by HPLC-HR-ESI-MS. The EIC for *m/z* 952.44 ± 0.01 is shown.

To address the possibility that the MeACC cluster produces a MeACC-containing peptide, the 59 kbp DNA fragment containing the MeACC cluster was cloned and introduced into a heterologous host strain, *S. lividans* TK23. HPLC-HR-ESI-MS analysis of the culture broth in the resulting transformant, TK23_MeACC (Table S1), revealed the production of a compound (*m/z* 952.445 [M + H]^+^) (Figure 3A), although the TK23_empty strain did not produce such a compound (Figure 3B). By the HR-ESI-MS data, the molecular formula, C_50_H_61_N_7_O_12_ (calculated for *m/z* 952.445 [M + H]^+^), was determined, and a database search with SciFinder suggested that the compound was Q6402A (4) (Figure 1D), which is produced by *Streptomyces* sp. Q-6402 [29]. As predicted, this compound was reported to be cyclic hexa-peptides, containing the MeACC building block. However, the amino-acid components (threonine, phenylalanine, glutamate, hydroxy-tryptophan, hydroxy phenylglycine, and 3) were, in part, different from our speculation based on the predicted substrate for the NRPS A domains (Table S6). We therefore tried to purify the compound from the TK23_MeACC strain and the original strain with the MeACC cluster to confirm the chemical structure. However, despite considerable efforts, the compound could not be obtained in these strains in amounts suitable for NMR analysis. Therefore, in the present study, we focused on the functional analysis of *orf29* and *orf30*, which should produce the MeACC building block.

### 3.3. Gene Inactivation of orf29 and orf30

To examine whether the *orf29* and *orf30* genes are involved in the biosynthesis of putative Q6402A, these two genes were inactivated by an in-frame deletion and introduced into a heterologous host strain, *S. lividans* TK23. The resulting transformants, TK23_MeACC_Δ*orf29* and TK23_MeACC_Δ*orf30*, were cultured and analyzed by HPLC-HR-ESI-MS. Compared to TK23_MeACC, carrying the MeACC cluster (wild type) (Figure 3A), the deletion of o*rf29* or *orf30* abolished the biosynthesis of putative Q6402A (Figure S3A–C). Thus, the *orf29* and *orf30* genes were found to have a major role in the biosynthesis of putative Q6402A. In a further HPLC-HR-ESI-MS analysis of the culture broth, the TK23_MeACC_Δ*orf29* strain was shown to produce a compound (*m/z* 938.428 [M + H]^+^) (Figure S3B) rather than putative Q6402A (*m/z* 952.445 [M + H]^+^). The mass decrease of 14 Da was in good agreement with the fact that the *orf29* gene homologous to radical SAM methyltransferase genes (Table S4) was inactivated; the Δ14 Da compound seemed to be a demethyl-Q6402A. Interestingly, production of the putative demethyl-Q6402A was also observed in the TK23_MeACC_Δ*orf30* strain*,* but only when 0.2% ACC was added to the culture medium (Figure S3D). These findings suggested that *orf29* did not catalyze *C*-methylation of the cyclopropane moiety of 2.

### 3.4. Feeding Experiments with ^13^C-Labeled L-Methionine in TK23_MeACC

In the biosynthesis of guangnanmycin (Figure 1C), compound 2 was produced from 1 by the catalysis of GnmY [11]. On the other hand, a Swb6/Swb7-mediated route from L-valine to 3 was proposed in the SW-163 biosynthesis [14] (Figure 1D). However, considering the fact that Swb6 and *orf30* share homology with GnmY (~40%), we expected that the precursor of 3 was also SAM (1) in the biosynthesis of SW-163C and Q6402A. To address this hypothesis, we performed feeding experiments using [1-^13^C]- or [5-^13^C]-L-methionine for the cultivation of TK23_MeACC, which produces putative Q6402A. HPLC-HR-ESI-MS analysis demonstrated that both ^13^C-labeled compounds were incorporated into the chemical structure of putative Q6402A (Figure S4). In particular, the incorporation of [1-^13^C]-L-methionine (*m/z* 953.447 [M + H]^+^, calculated for 953.447) suggested that the origin of the MeACC moiety in Q6402A is 1, which is derived from L-methionine. The feeding of [5-^13^C]-L-methionine resulted in the production of a labeled putative Q6402A (*m/z* 954.451 [M+H]^+^). The molecular weight change of 2 Da was also in agreement with the fact that Q6402A possesses two methyl groups (Figure 1D): an *N*-methyl group on the phenylalanine residue and a *C*-methyl group on the ACC residue (namely, MeACC). This finding also suggested that these two methyl groups are derived from L-methionine via SAM (1). The *N*-methylation would be performed by the methyltransferase domain in *orf22* (NRPS) (Figure 2).

### 3.5. Heterologous Co-expression of the orf29 and orf30 Genes in E. coli

Based on the finding that [1-^13^C]-L-methionine was incorporated into putative Q6402A, compound 3 was expected to be derived from 1. We therefore carried out a heterologous co-expression experiment using the *orf29* and *orf30* genes to confirm that these two genes are involved in the biosynthesis of 3. As mentioned earlier, *orf29* shares homology with a B12-binding domain-containing radical SAM protein. Therefore, we expected that reconstitution of the [4Fe − 4S]^+^ cluster in *E. coli* was required for the expression of *orf29* in an active form. For this reason, we employed *E. coli* C41 harboring pRKSUF017 (EcSUF), in which the SUF operon genes are expressed (Table S1) [21]. The co-expression strain, EcSUF_*orf29*_*orf30* (Table S1), was cultured, and we examined whether or not the strain produced 3 by HPLC-HR-ESI-MS analysis. Whereas, the EcSUF_*orf29* strain that expresses only the *orf29* gene did not produce 3 and the co-expression strain EcSUF_*orf29*_*orf30* did produce 3 (Figure S5A–D). These findings demonstrated that *orf29* and *orf30* produce 3 in a synergistic manner. However, interestingly, compound 3 was not produced by the EcSUF_*orf29* in the culture medium supplemented with 2 (Figure S5E).

The HPLC retention time of 3 was superimposable to that of a chemically synthesized standard, (*1R*,*2R*)-MeACC (5), rather than (*1S*,*2R*)-MeACC (**6**) (Figure S5A–C), suggesting that the absolute configuration of 3 is *1R*,*2R* or its enantiomer (*1S,2S*) (see below, Section 3.7).

### 3.6. Functional Analysis of orf30 In Vitro

**Figure 4:**
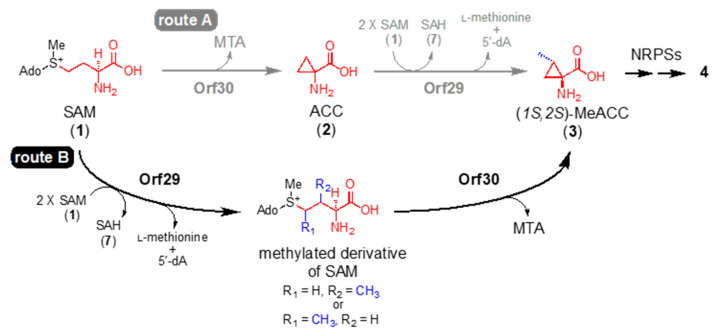
Biosynthetic routes from *S*-adenosyl-L-methionine (SAM) to MeACC in vitro. Two biosynthetic routes to 3, route A and route B, were proposed in this study.

**Figure 5:**
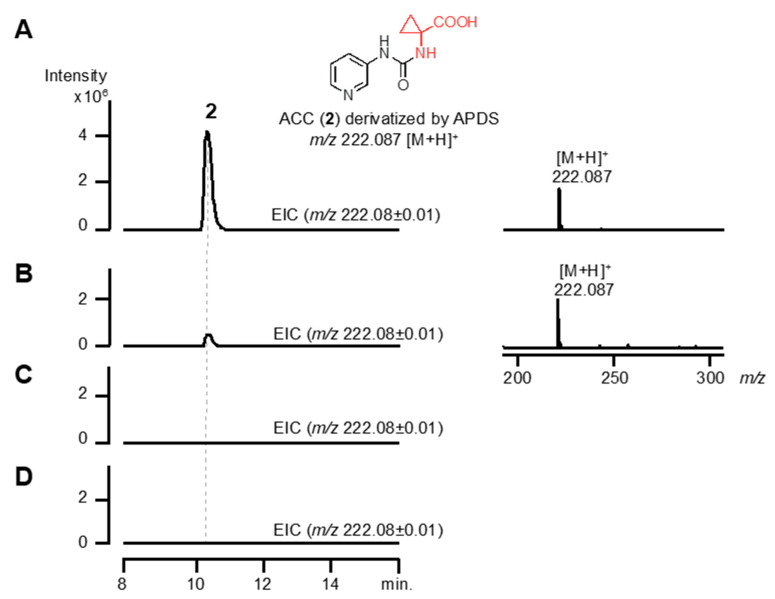
Enzyme reaction of rOrf30 in vitro. Compound 2 derivatized by 3-aminopyridinyl-*N*-hydroxysuccinimidyl carbamate (APDS) was used as a standard and analyzed by HPLC-HR-ESI-MS (**A**) Compound 1 or (**B**) L-methione (**C**) was incubated with rOrf30, and then the reaction product was derivatized by APDS and analyzed by HPLC-HR-ESI-MS. (**D**) Compound 1 was incubated in a reaction mixture without rOrf30, and then the reaction mixture was derivatized by APDS and analyzed by HPLC-HR-ESI-MS.

*orf30* shares identity with GmnY (40%) (Table S5). We therefore speculated that *orf30* also catalyzes the formation of 2 from 1 (route A; Figure 4). To confirm the cyclopropanation reaction by *orf30*, the recombinant enzyme (rOrf30) was used for enzyme assays in vitro, and we performed an enzyme reaction using 1 as a substrate. Predictably, HPLC-HR-ESI-MS analysis of the reaction mixture revealed that rOrf30 enzymatically converted 1 into 2, while an enzyme reaction employing a heat-denatured enzyme did not produce 2 (Figure 5). On the other hand, an enzyme reaction using L-methionine as a substrate did not give any enzyme product (Figure 5). Thus, these results demonstrated that *orf30* is a bacterial ACC synthase, catalyzing the ACC formation from 1.

Although *orf30* was found to be the second example of bacterial ACC synthase, *orf30* had only 40% similarity with GnmY (Table S5). We therefore investigated the enzymatic properties of rOrf30. Gel-filtration chromatography analysis demonstrated that the native rOrf30 was homodimeric (Figure S6A). The optimum pH was found to be 8 (Figure S6B), and rOrf30 showed the highest activity at 30 °C in the enzyme reaction (Figure S6C). To our surprise, rOrf30 had an apparent *K*_m_ of 1.09 ± 0.33 mm for 1 and *K*_cat_ of 0.0049 ± 0.00068 s^−1^ (Figure S6D), while it was reported that GnmY exhibited an apparent *K*_m_ of 21.6 ± 1.8 µM for 1 and *K*_cat_ of 0.79 ± 0.02 s^−1^. Thus, the *K*_cat_ value of rOrf30 was about 1,900 times lower than that of GnmY, suggesting that SAM (1) is not a genuine substrate of *orf30*.

### 3.7. Functional Analysis of rOrf29 In Vitro

Based on the results from the *orf29* gene knockout (Section 3.3), the co-expression experiments (Section 3.5), and the kinetic study of rOrf30 (Section 3.6), the methyl group of MeACC (3) seemed to be introduced prior to the cyclopropanation step catalyzed by *orf30*. We therefore hypothesized that *orf29* catalyzes the methylation of 1, and then the resulting methylated derivative of SAM is converted into 3 by *orf30* (route B; Figure 4). To examine this hypothesis, the recombinant *orf29* (rOrf29) was overexpressed in *E. coli* (Figure S7A). As mentioned earlier, *orf29* was expected to be a radical SAM methyltransferase, containing a B12-binding domain. Therefore, we employed the *E. coli* EcSufBtu strain (Table S1), which expresses the *suf* [21] and *btu* [22] operons. After the reconstitution of the iron-sulfur cluster in rOrf29 (Figure S7B), rOrf29 was used for enzyme assays in vitro.

**Figure 6:**
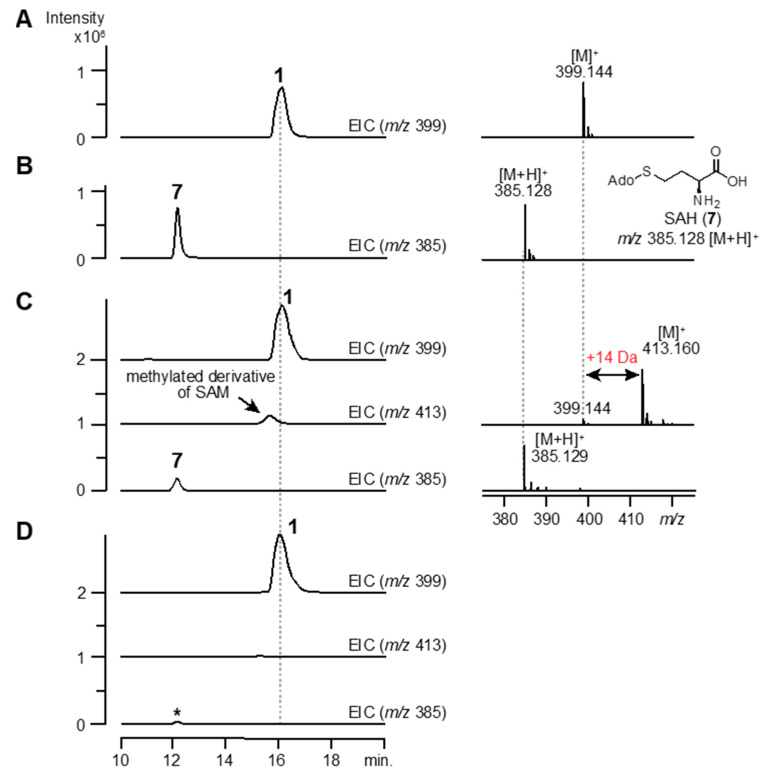
Enzyme reaction of rOrf29 *in vitro*. Standard compounds, SAM (1) (**A**) and *S*-adenosyl-L-homocysteine (SAH) (7) (**B**), were analyzed by HPLC-HR-ESI-MS. Compound 1 was incubated with (**C**) or without (**D**), the reconstituted rOrf29, and then the reaction mixtures were analyzed by HPLC-HR-ESI-MS. EICs for 1 (*m*/*z* 399), **7** (*m*/*z* 385) and the methylated derivative of SAM (*m*/*z* 413) are shown. The asterisk denotes a spontaneously produced SAH (7).

The reconstituted rOrf29 was reacted with SAM (1) (*m/z* 399.144 [M]^+^, calculated for 399.144) under anaerobic conditions, and the reaction product was analyzed by HPLC-HR-ESI-MS. As expected, rOrf29 produced a compound with a 14 Da increase (*m*/*z* 413.160 [M]^+^), which was thought to be a methylated derivative of SAM (Figure 6). Moreover, *S*-adenosyl homocysteine (SAH) (7) (Figure 6) and 5′-deoxyadenosine (5′-dA) (Figure S8) were detected in the enzyme reaction. An enzyme reaction without rOrf29 did not yield these compounds (Figure 6 and Figure S8). On the other hand, an enzyme reaction with compound 2 did not yield compound 3 (Figure S9D). These results implied that rOrf29 recognizes not 2 but 1 for *C*-methylation. To examine if the methylated derivative of 1 is converted into 3, rOrf30 was added to an rOrf29 reaction mixture that had been incubated with 1 (Figure S9E). As expected, this stepwise enzyme reaction produced 3 (Figure S9E). The absolute configuration of the enzymatically produced 3 was determined to be (*1S,2S*)-MeACC by the advanced Marfey’s method (Figure S10).

## 4. Discussion

In this study, from *Streptomyces* genomes in our lab stock strains, we identified two genes, *orf29* and *orf30*, which are involved in the biosynthesis of MeACC (3). At the beginning of the study, we hypothesized that 3 is produced from SAM (1) via ACC (2) (route A; Figure 4), because *orf30* shared similarity (40%) with the bacterial ACC synthase GnmY, which catalyzes the cyclopropanation using 1 as the substrate (Figure 1C). In fact, we detected the enzyme activity, but the kinetic data of rOrf30 were quite different from those of GmnY. Furthermore, our phylogenetic analysis suggested that *orf30* and GnmY formed a distinct clade (Figure S2). These unexpected differences allowed us to think about an alternative biosynthetic route from 1 to 3 (route B; Figure 4). Such a route was also supported by the following observations from the in vivo experiments: 1) the productivity of the putative demethyl-Q6402A was observed in the TK23_MeACC_Δ*orf30* strain, only when 0.2% ACC was added to the culture medium (Figure S3D). 2) MeACC (3) was not produced by the EcSUF_*orf29* strain in the culture medium supplemented with AAC (2) (Figure S5E). To confirm the alternative biosynthetic route (route B) from 1 to 3 (Figure 4), we investigated the catalytic function of the reconstituted radical SAM enzyme, rOrf29. The in vitro analysis clearly demonstrated that SAM (1) was converted into the methylated derivative by rOrf29 (Figure 6C) and was then converted into MeACC (3) by the rOrf30 catalysis (Figure S9E). Because rOrf30 converts SAM (1) to ACC (2) with the low reaction rate, the methylated derivative of SAM produced by rOrf29 seems to be a better substrate of rOrf30. From these findings, we concluded that the rOrf29-mediated *C*-methylation occurs in the L-methionine moiety of SAM (1) prior to the cyclopropanation reaction catalyzed by rOrf30. The production of (*1S,2S*)-MeACC revealed that the *C*-methylation of 1 is a stereospecific reaction by rOrf29. To determine the *C*-methylation position (C3 or C4) of this unique radical, the methylated derivative of SAM produced by rOrf29, we now investigate biochemical properties of the SAM enzyme to improve the reaction efficiency to isolate the product for structural determination.

Based on a database search, *orf29* was found to have an iron-sulfur cluster-binding domain and a cobalamin-binding domain. Therefore, *orf29* belongs to the class B methyltransferase group in radical SAM methyltransferases. Class B enzymes are the most abundant radical SAM methyltransferases and appear to be the most versatile, catalyzing the methylation of both unactivated carbon and phosphorus centers [22]. For example, CloN6 (ID no. RS42) [30], Fms7 (ID no. RS45) [31], and GenK (ID no. RS47) (Table S4) [32] catalyze the methylation of unactivated carbon. PhpK (ID no. RS50) [33] mediates *P*-methylation. In the phylogenetic analysis, *orf29* was found to form a distinct clade with these class B methyltransferases. In addition, to date, there has been no report of a class B enzyme that methylates 1. Thus, *orf29* is a novel radical SAM methyltransferase, catalyzing *C*-methylation of the L-methionine moiety of SAM (**1**) (Figure 4).

We found that the *orf29* homologues (RS1 to RS6) and the *orf30* homologues (AS1 to AS6) are positioned next to each other in their gene cluster (Figure S11), strongly suggesting that these six gene clusters are responsible for the production of peptide natural products, containing the MeACC building block (Figure S11). In fact, the gene cluster containing RS1(*swb7*)-AS1(*swb6*) was reported to produce SW-163C [14] (Figure 1D and S11B). In addition, retimycin A, which is an SW-163C analogue compound, is also produced by the gene cluster with RS2-AS2 [34] (Figure S11C). In this study, we showed that the MeACC cluster from *S.* *violaceoniger* 4521-SVS3 (Figure 2 and Figure S11A) is involved in the biosynthesis of putative Q6402A (C_50_H_61_N_7_O_12_, calculated for *m/z* 952.445 [M + H]^+^) (Figure 3). On the other hand, *Streptomyces* sp. Q6402, which is the original strain producing Q6402A [29], was also reported to produce an analogue compound Q6402B (C_51_H_63_N_7_O_12_, calculated for *m/z* 965.453 [M + H]^+^) with a longer fatty acid side chain (6-methylheptanoic acid). It is possible that the MeACC cluster from *S.* *violaceoniger* 4521-SVS3 also produces putative Q6402B, because we observed the production of not only putative demethyl-Q6402A but also putative demethyl-Q6402B in the TK23_MeACC_Δ*orf29* strain (Figure S3B and S3D). Furthermore, the production of the demethyl compounds suggested that the A domain of *orf23* (NRPS) would accept 2 as a substrate in addition to 3.

## 5. Conclusions

In this study, we identified two new enzymes, *orf29* and *orf30*, which are the B12-binding domain-containing radical SAM methyltransferase and the bacterial ACC synthase, respectively. In vitro, rOrf29 was found to catalyze the *C*-methylation of the L-methionine moiety of SAM (1). The rOrf29 reaction product was then converted into MeACC (3) by rOrf30. Thus, we demonstrate that *C*-methylation of SAM occurs prior to cyclopropanation in the biosynthesis of a bacterial MeACC (norcoronamic acid).

## Supplementary Materials

The following are available online at https://www.mdpi.com/2218-273X/10/5/775/s1, Figure S1: Phylogenetic analysis of the amino acid sequences from Orf29 and its homologues, Figure S2: Phylogenetic analysis of the amino acid sequences from Orf30 and its homologues, Figure S3: Gene inactivation of *orf29* and *orf30*, Figure S4: Feeding experiments using ^13^C-labeled L-methionine, Figure S5: Heterologous coexpression of the *orf29* and *orf30* genes in *E. coli*, Figure S6: Enzymatic properties of rOrf30, Figure S7: Overexpression of rOrf29 and reconstitution of the iron-sulfur cluster, Figure S8: Detection of Ado-CH_3_ in the rOrf29 reaction, Figure S9: Enzymatic synthesis of MeACC (3) by a sequential reaction with rOrf29 and rOrf30, Figure S10: Determination of the absolute configuration of MeACC (3), Figure S11: MeACC cluster homologues, Table S1: Plasmids and strains used in this study, Table S2: Primers used in this study, Table S3: Sequences of the synthetic DNA fragments for construction of the plasmid pBAD24_BtuCEDFB, Table S4: Orf29 homologs used in the phylogenetic analysis, Table S5: Orf30 homologs used in the phylogenetic analysis, Table S6: Substrate specificity analysis of six A domains in the MeACC cluster.

## References

[1] Marahiel M.A., Stachelhaus T., Mootz H.D. (1997). Modular peptide synthetases involved in nonribosomal peptide synthesis. Chem. Rev..

[2] Mootz H.D., Schwarzer D., Marahiel M.A. (2002). Ways of assembling complex natural products on modular nonribosomal peptide synthetases. ChemBioChem..

[3] Schwarzer D., Finking R., Marahiel M.A. (2003). Nonribosomal peptides: From genes to products. Nat. Prod. Rep..

[4] Walsh C.T., O’Brien R.V., Khosla C. (2013). Nonproteinogenic amino acid building blocks for nonribosomal peptide and hybrid polyketide scaffolds. Angew. Chem. Int. Ed. Engl..

[5] Kudo F., Miyanaga A., Eguchi T. (2019). Structural basis of the nonribosomal codes for nonproteinogenic amino acid selective adenylation enzymes in the biosynthesis of natural products. J. Ind. Microbiol. Biotechnol..

[6] Hedges J.B., Ryan K.S. (2020). Biosynthetic Pathways to Nonproteinogenic alpha-Amino Acids. Chem. Rev..

[7] Thibodeaux C.J., Chang W.C., Liu H.W. (2012). Enzymatic chemistry of cyclopropane, epoxide, and aziridine biosynthesis. Chem. Rev..

[8] Wessjohann L.A., Brandt W., Thiemann T. (2003). Biosynthesis and metabolism of cyclopropane rings in natural compounds. Chem. Rev..

[9] Adams D.O., Yang S.F. (1979). Ethylene biosynthesis: Identification of 1-aminocyclopropane-1-carboxylic acid as an intermediate in the conversion of methionine to ethylene. Proc. Natl. Acad. Sci. USA..

[10] Zha L., Jiang Y.D., Henke M.T., Wilson M.R., Wang J.X., Kelleher N.L., Balskus E.P. (2017). Colibactin assembly line enzymes use S-adenosylmethionine to build a cyclopropane ring. Nat. Chem. Biol..

[11] Xu Z., Pan G., Zhou H., Shen B. (2018). Discovery and Characterization of 1-Aminocyclopropane-1-carboxylic Acid Synthase of Bacterial Origin. J. Am. Chem. Soc..

[12] Kurosawa K., Takahashi K., Tsuda E. (2001). SW-163C and E, novel antitumor depsipeptides produced by Streptomyces sp. I. Taxonomy, fermentation, isolation and biological activities. J. Antibiot. (Tokyo).

[13] Takahashi K., Koshino H., Esumi Y., Tsuda E., Kurosawa K. (2001). SW-163C and E, novel antitumor depsipeptides produced by Streptomyces sp. II. Structure elucidation. J. Antibiot. (Tokyo).

[14] Watanabe K., Hotta K., Nakaya M., Praseuth A.P., Wang C.C., Inada D., Takahashi K., Fukushi E., Oguri H., Oikawa H. (2009). Escherichia coli allows efficient modular incorporation of newly isolated quinomycin biosynthetic enzyme into echinomycin biosynthetic pathway for rational design and synthesis of potent antibiotic unnatural natural product. J. Am. Chem. Soc..

[15] Hercouet A., Godbert N., Le Corre M. (1998). Enantiospecific synthesis of norcoronamic acids. Tetrahedron-Asymmetr.

[16] Hercouet A., Bessières B., Le Corre M. (1996). Expedient synthesis of (−)-(1S, 2R)-Allonorcoronamic acid. Tetrahedron: Asymmetry..

[17] Blin K., Shaw S., Steinke K., Villebro R., Ziemert N., Lee S.Y., Medema M.H., Weber T. (2019). antiSMASH 5.0: Updates to the secondary metabolite genome mining pipeline. Nucleic. Acids. Res..

[18] Komatsu M., Komatsu K., Koiwai H., Yamada Y., Kozone I., Izumikawa M., Hashimoto J., Takagi M., Omura S., Shin-ya K. (2013). Engineered Streptomyces avermitilis host for heterologous expression of biosynthetic gene cluster for secondary metabolites. ACS. synthetic biology.

[19] Kieser T., Bibb M.J., Buttner M.J., Chater K.F., Hopwood D.A. (2000). Practical streptomyces genetics.

[20] Gust B., Challis G.L., Fowler K., Kieser T., Chater K.F. (2003). PCR-targeted Streptomyces gene replacement identifies a protein domain needed for biosynthesis of the sesquiterpene soil odor geosmin. Proc. Natl. Acad. Sci. USA.

[21] Takahashi Y., Tokumoto U. (2002). A third bacterial system for the assembly of iron-sulfur clusters with homologs in archaea and plastids. J. Biol. Chem..

[22] Lanz N.D., Blaszczyk A.J., McCarthy E.L., Wang B., Wang R.X., Jones B.S., Booker S.J. (2018). Enhanced Solubilization of Class B Radical S-Adenosylmethionine Methylases by Improved Cobalamin Uptake in Escherichia coli. Biochemistry.

[23] Guzman L.M., Belin D., Carson M.J., Beckwith J. (1995). Tight regulation, modulation, and high-level expression by vectors containing the arabinose PBAD promoter. J. Bacteriol..

[24] Blaszczyk A.J., Wang R.X., Booker S.J. (2017). TsrM as a Model for Purifying and Characterizing Cobalamin-Dependent Radical S-Adenosylmethionine Methylases. Methods Enzymol..

[25] Fujii K., Ikai Y., Oka H., Suzuki M., Harada K. (1997). A nonempirical method using LC/MS for determination of the absolute configuration of constituent amino acids in a peptide: Combination of Marfey’s method with mass spectrometry and its practical application. Anal. Chem..

[26] Fujii K., Ikai Y., Mayumi T., Oka H., Suzuki M., Harada K. (1997). A nonempirical method using LC/MS for determination of the absolute configuration of constituent amino acids in a peptide: Elucidation of limitations of Marfey’s method and of its separation mechanism. Anal. Chem..

[27] Rottig M., Medema M.H., Blin K., Weber T., Rausch C., Kohlbacher O. (2011). NRPSpredictor2—A web server for predicting NRPS adenylation domain specificity. Nucleic. Acids. Res..

[28] Hubbard B.K., Thomas M.G., Walsh C.T. (2000). Biosynthesis of L-p-hydroxyphenylglycine, a non-proteinogenic amino acid constituent of peptide antibiotics. Chem. Biol..

[29] Hiramoto M., Niwa A., Miyake A., Yamamoto H., Takebayashi Y., Nishikawa T., Shibazaki M., Nagai K. (1993). Structures of novel phospholipase A2 inhibitors, Q-6402-A and B. Tennen Yuki Kagobutsu Toronkai Koen Yoshishu.

[30] Westrich L., Heide L., Li S.M. (2003). CloN6, a novel methyltransferase catalysing the methylation of the pyrrole-2-carboxyl moiety of clorobiocin. ChemBioChem..

[31] Kuzuyama T., Seki T., Dairi T., Hidaka T., Seto H. (1995). Nucleotide sequence of fortimicin KL1 methyltransferase gene isolated from Micromonospora olivasterospora, and comparison of its deduced amino acid sequence with those of methyltransferases involved in the biosynthesis of bialaphos and fosfomycin. J. Antibiot. (Tokyo).

[32] Kim H.J., McCarty R.M., Ogasawara Y., Liu Y.N., Mansoorabadi S.O., LeVieux J., Liu H.W. (2013). GenK-catalyzed C-6′ methylation in the biosynthesis of gentamicin: Isolation and characterization of a cobalamin-dependent radical SAM enzyme. J. Am. Chem. Soc..

[33] Werner W.J., Allen K.D., Hu K., Helms G.L., Chen B.S., Wang S.C. (2011). In vitro phosphinate methylation by PhpK from Kitasatospora phosalacinea. Biochemistry.

[34] Duncan K.R., Crusemann M., Lechner A., Sarkar A., Li J., Ziemert N., Wang M., Bandeira N., Moore B.S., Dorrestein P.C. (2015). Molecular networking and pattern-based genome mining improves discovery of biosynthetic gene clusters and their products from Salinispora species. Chem. Biol..

