# Supplementary material for: C-Methylation of S-adenosyl-L-Methionine Occurs Prior to Cyclopropanation in the Biosynthesis of 1-Amino-2-Methylcyclopropanecarboxylic Acid (Norcoronamic Acid) in a Bacterium
Source: Biomolecules. 2020 May 16;10(5):775. doi: 10.3390/biom10050775 (PMC7277169; doi:10.3390/biom10050775)
Supplement: Supplementary file 1 [file biomolecules-10-00775-s001.pdf]

C-Methylation of *S*-adenosyl-L-methionine occurs prior to cyclopropanation in the biosynthesis of a bacterial 1-amino-2-methylcyclopropanecarboxylic acid (norcoronamic acid).

(Supplementary Materials)

Chitose Maruyama [1,†], Yukiko Chinone [1,†], Shusuke Sato [2], Fumitaka Kudo [2], Kosuke Ohsawa [3], Junya Kubota [3], Junko Hashimoto [4], Ikuko Kozone [4], Takayuki Doi [3], Kazuo Shin-ya [5-7], Tadashi Eguchi [2], and Yoshimitsu Hamano[1,*]

1 Department of Bioscience, Fukui Prefectural University, 4-1-1 Yoshida-Gun, Fukui 910-1195, Japan; c-maruyama@fpu.ac.jp (C.M.); s1873013@g.fpu.ac.jp (Y.C.)
2 Department of Chemistry, Tokyo Institute of Technology, 2-12-1 O-okayama, Meguro-ku, Tokyo 152-8551, Japan; satou.s.ae@m.titech.ac.jp (S.S.); fkudo@chem.titech.ac.jp (F.K.)
3 Graduate School of Life Sciences, Tohoku University, 6-3 Aza-aoba, Aramaki, Aoba-ku, Sendai 980-8578, Japan; kosuke@mail.pharm.tohoku.ac.jp (K.O.); junya.kubota.s7@gmail.com (J.K.); doi_taka@mail.pharm.tohoku.ac.jp (T.D.)
4 Japan Biological Informatics Consortium (JBIC), 2-4-7 Aomi, Koto-ku, Tokyo 135-0064, Japan; junko.hashimoto@aist.go.jp (J.H.); ikuko-kozone@aist.go.jp (I.K.)
5 National Institute of Advanced Industrial Science and Technology, 2-4-7 Aomi, Koto-ku, Tokyo 135-0064, Japan; k-shinya@aist.go.jp (K.S.)
6 The Biotechnology Research Center, The University of Tokyo, 1-1-1 Yayoi, Bunkyo-ku, Tokyo 113-8657, Japan
7 Collaborative Research Institute for Innovative Microbiology, The University of Tokyo, 1-1-1 Yayoi, Bunkyo-ku, Tokyo, 113-8657, Japan

† These authors contributed equally to this work.
* Correspondence: hamano@fpu.ac.jp; Tel.: +81-776-61-6000 (Y.H.)

1. Supplementary Figures (Figure S1 – S11)

2. Supplementary Tables (Tables S1 – S6)

1. Supplementary Figures

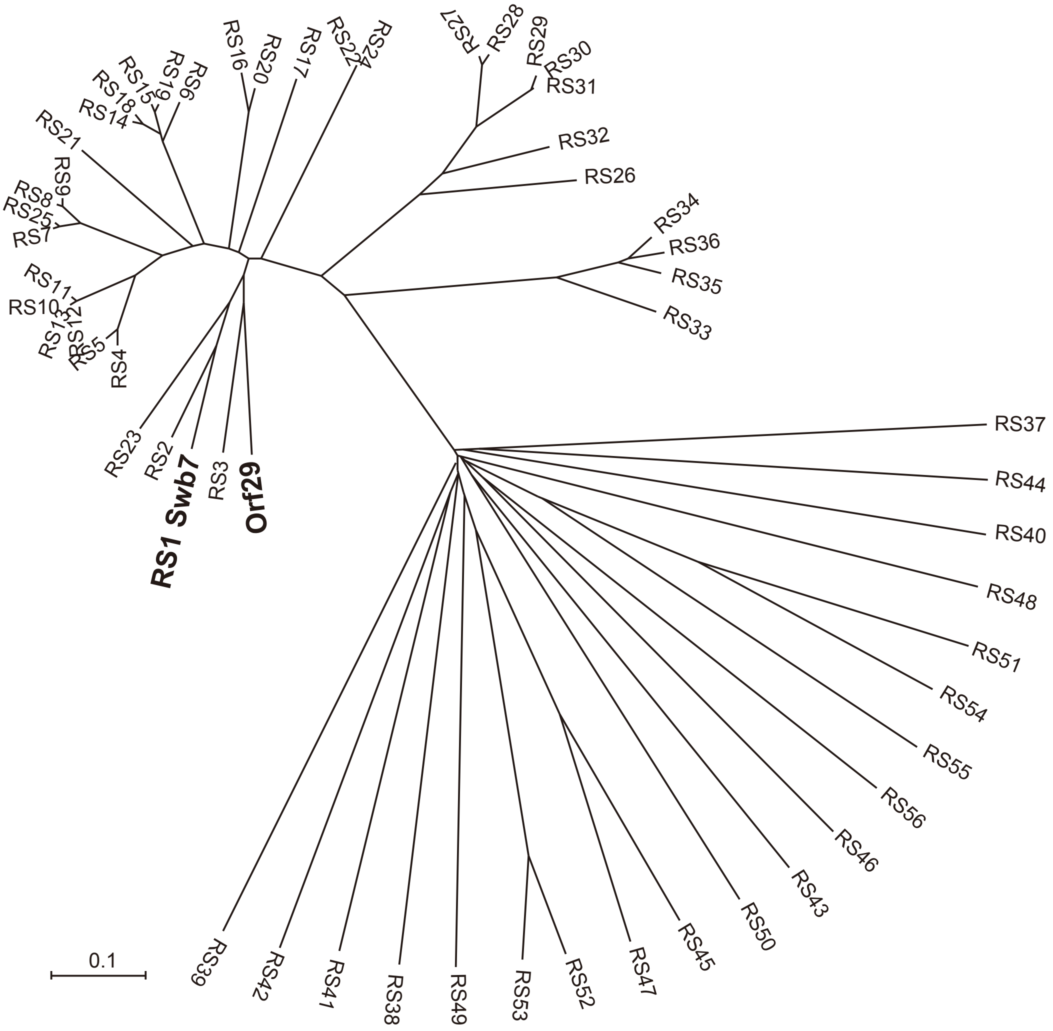

Figure S1. Phylogenetic analysis of the amino acid sequences from Orf29 and its homologues. The phylogenetic tree was generated by MEGA7 using the maximum likelihood method with a bootstrap test of 1,000 replicates. The information about the homologue enzymes are listed in Table S3.

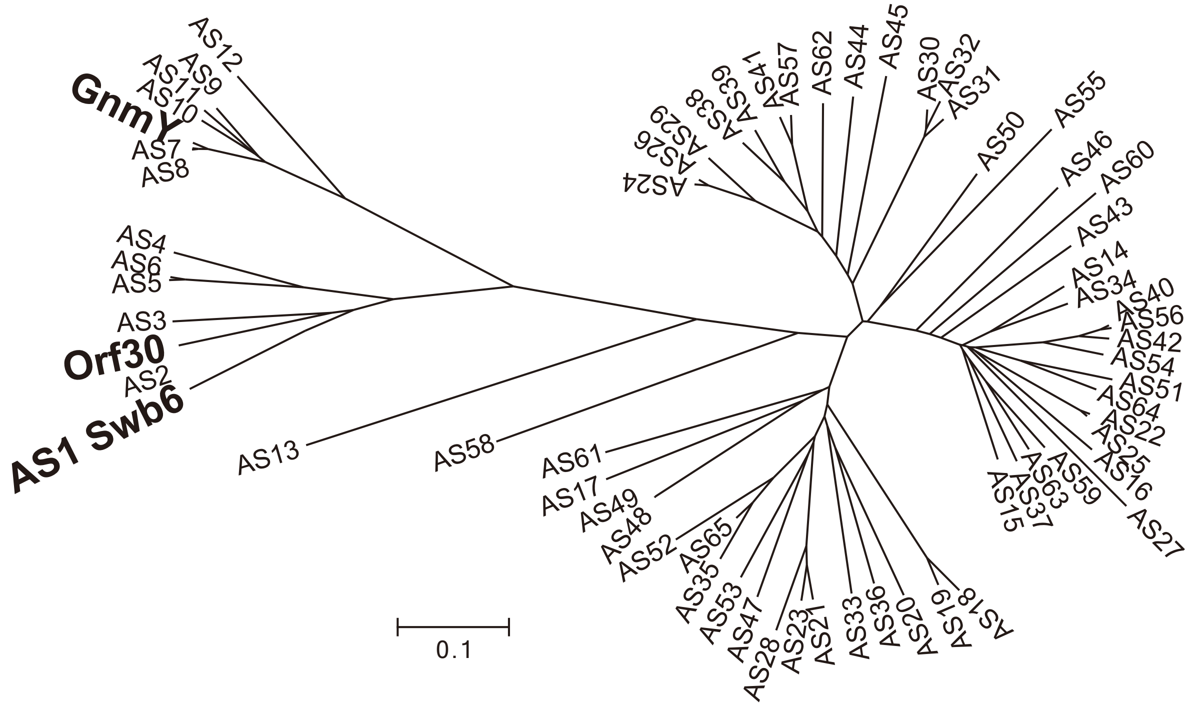

Figure S2. Phylogenetic analysis of the amino acid sequences from Orf30 and its homologues. The phylogenetic tree was generated by MEGA7 using the maximum likelihood method with a bootstrap test of 1,000 replicates. The information about the homologue enzymes are listed in Table S4.

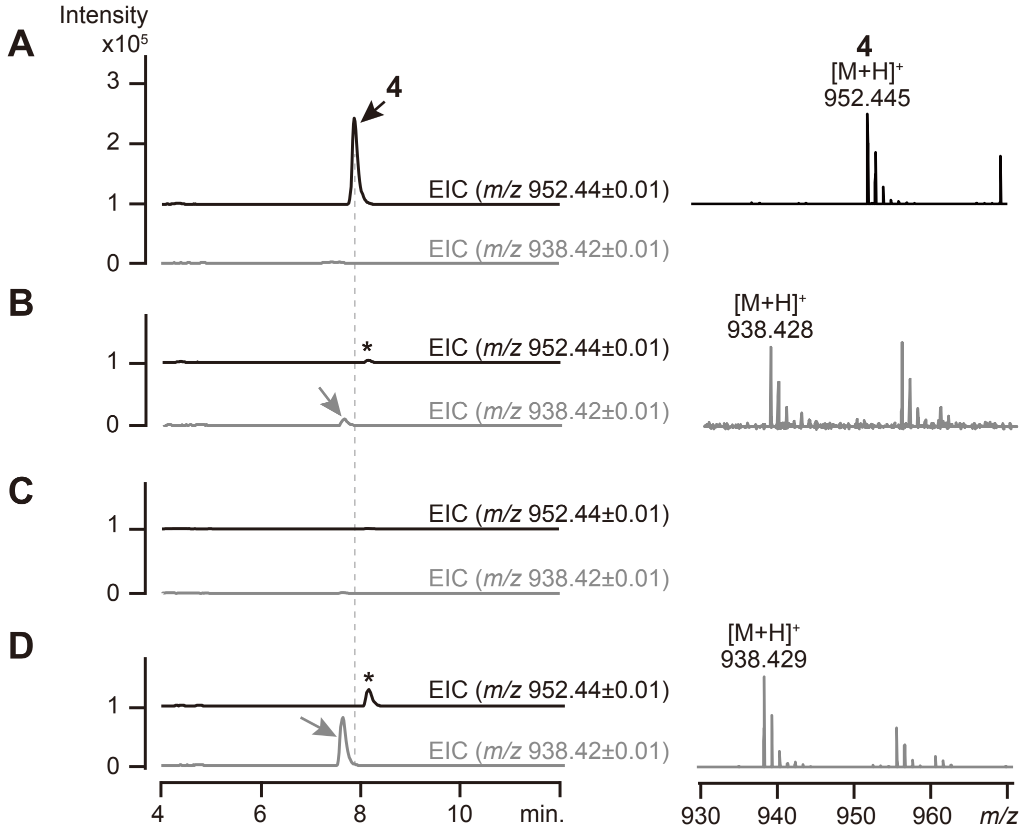

Figure S3. Gene inactivation of *orf29* and *orf30*. (**A**) The MeACC cluster was introduced into a heterologous host strain, *S. lividans* TK23. The resulting transformants, TK23_MeACC, was cultured and analyzed by HPLC-HR-ESI-MS (The data was identical to Figure 3A). The *orf29* (**B**) and *orf30* (**C**) genes were inactivated and introduced into a heterologous host strain, *S. lividans* TK23. The resulting transformants, TK23_MeACC_Δ*orf29* and TK23_MeACC_Δorf*30*, were cultured and analyzed by HPLC-HR-ESI-MS. (**D**) TK23_MeACC_Δorf*30*, were cultured with the medium supplemented with 0.2% (w/v) ACC (**2**) and analyzed by HPLC-HR-ESI-MS. Extracted ion chromatograms (EICs) for *m/z* 952.44±0.01 and *m/z* 938.42±0.01 are shown. The asterisks denote a putative demethyl-Q6402B (see the Discussion section).

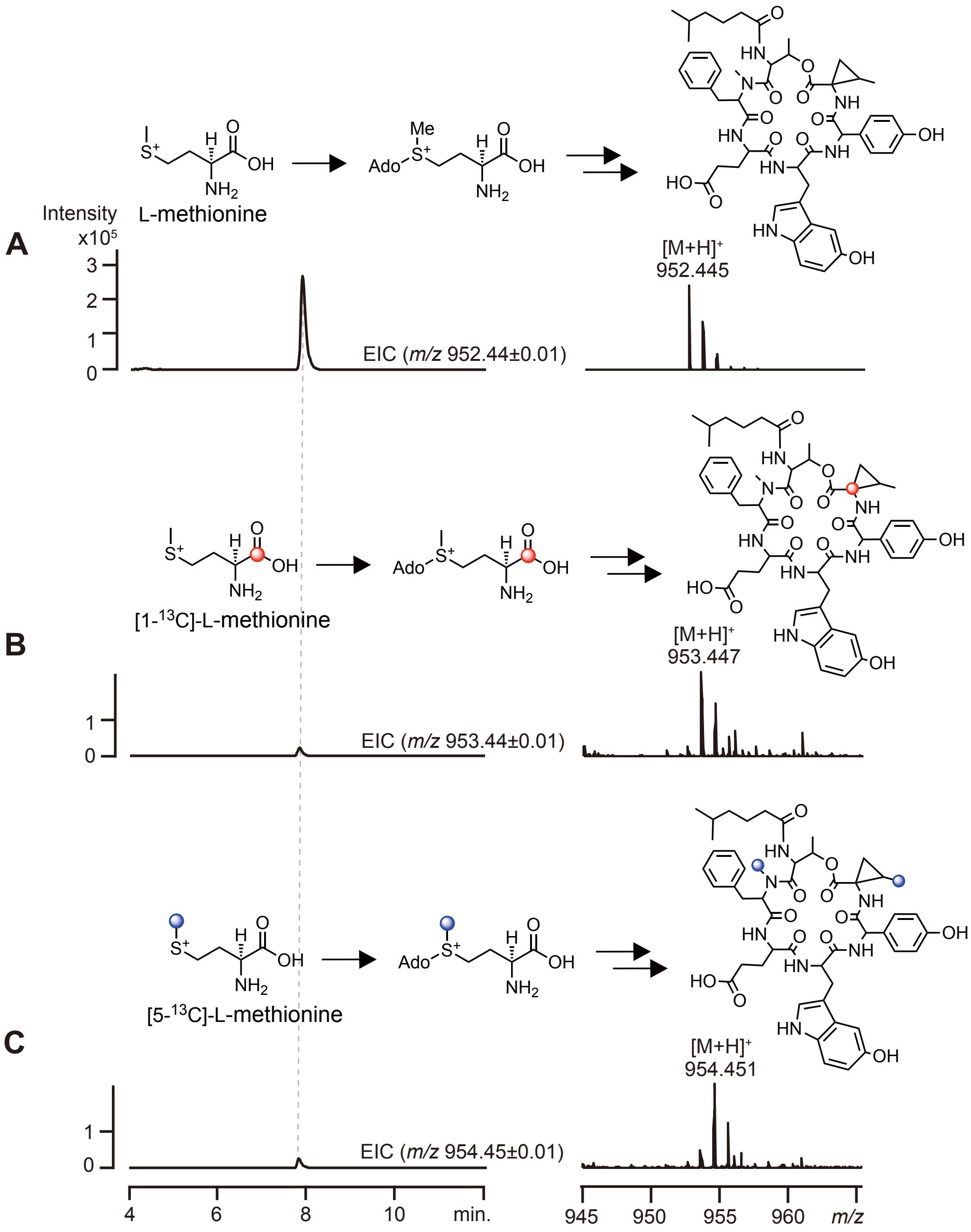

Figure S4. Feeding experiments using ^{13}C-labeled L-methionine. The TK23_MeACC strain was cultured with 2×SK No.2 medium (**A**), 2×SK No.2 medium supplemented with 0.1% [1-^{13}C]-L-methionine (labeled carbon is shown in red) (**B**), and 2×SK No.2 medium supplemented with 0.1% [5-^{13}C]-L-methionine (labeled carbon is shown in blue) (**C**). The culture broths were analyzed by HPLC-HR-ESI-MS. EICs for *m/z* 952.44±0.01, 953.44±0.01, 954.45±0.01 are shown.

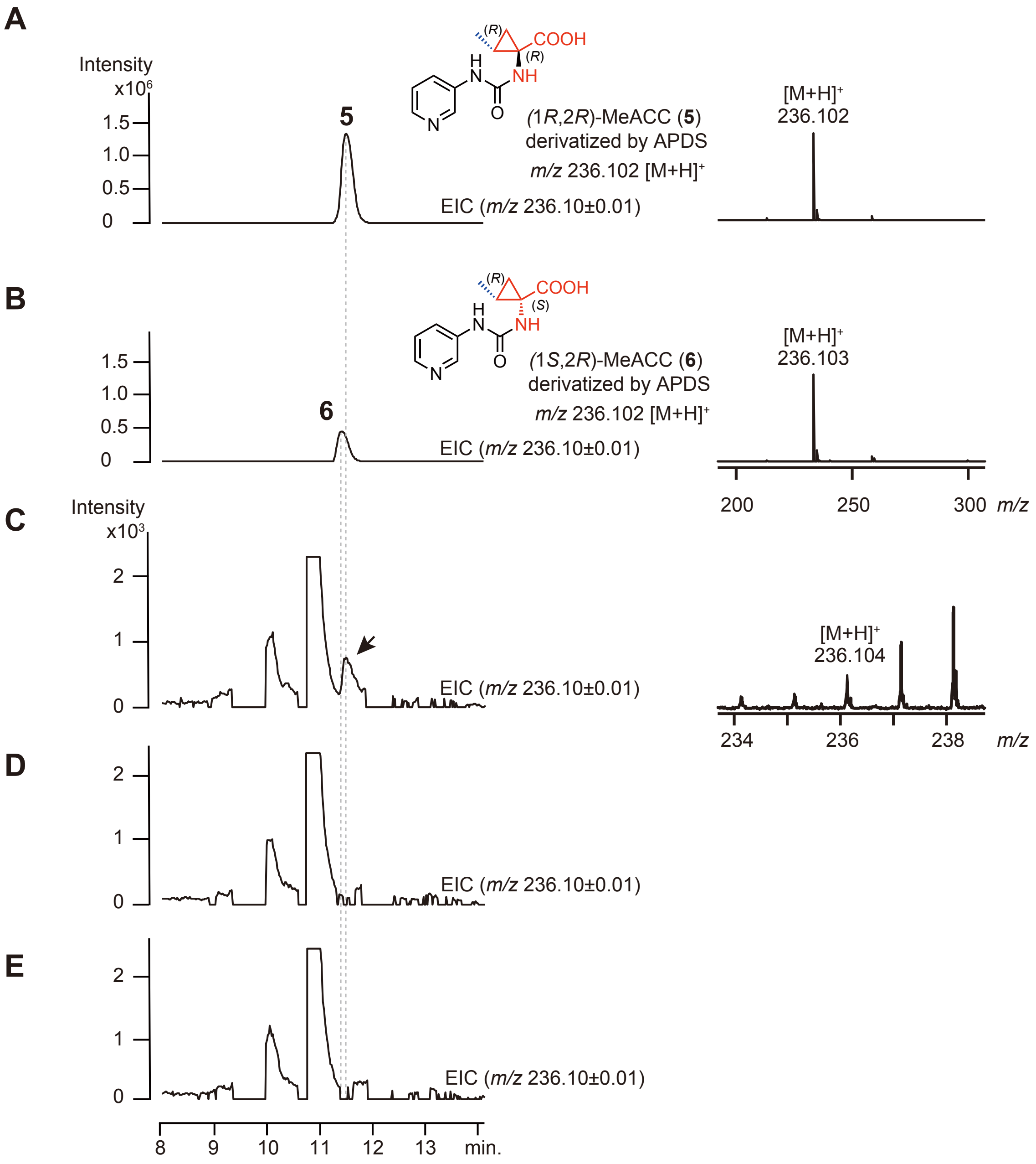

Figure S5. Heterologous coexpression of the *orf29* and *orf30* genes in *E. coli*. Chemically synthesized compounds, (*1R,2R*)-MeACC (**5**) (**A**) and (*1S,2R*)-MeACC (**6**) (**B**) , were used as standards and analyzed by HPLC-HR-ESI-MS. The culture broths from EcSuf_*orf29_orf30* (**C**) and EcSuf_*orf29* (**D**) were analyzed by HPLC-HR-ESI-MS. (**E**) The EcSuf_*orf29* strain was grown in a medium supplemented with 0.2% (w/v) ACC, and its culture broth was analyzed by HPLC-HR-ESI-MS. EICs for *m/z* 236.10±0.01 are shown.

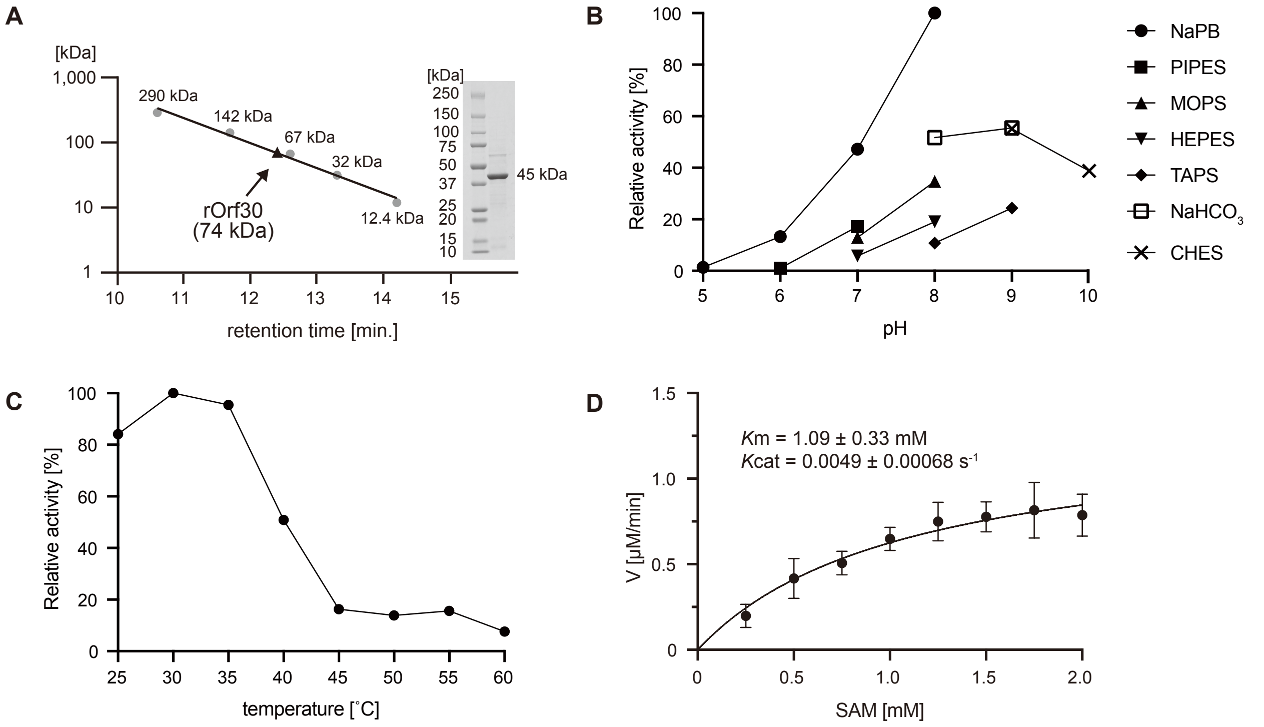

Figure S6. Enzymatic properties of rOrf30. (**A**)The purified rOrf30 was subjected to SDS-PAGE and was then stained with CBB R-250. The native molecular mass of rOrf30 was estimated by gel-filtration chromatography using SunSec diol-30 (4 µm, 4.6 × 300 mm, ChromaNik Technologies) at 25 °C at a flow rate of 0.3 mL/min and with 50 mM NaPB (pH 6.8) containing 300 mM NaCl run over 30 min. Glutamate dehydrogenase (290 kDa), lactate dehydrogenase (142 kDa), enolase (67 kDa), myokinase (32 kDa), and cytochrome C (12.4 kDa) (Oriental Yeast) were used as the standard molecular masses. (**B**) The optimum pH for the rOrf30 enzyme reaction was determined using different range of pH buffers: NaPB (pH 5 to 8), PIPES (pH 6 and 7), MOPS (pH 7 and 8), HEPES (pH 7 and 8), TAPS (pH 8 and 9), $NaHCO_3$ buffer (pH 8 and 9), and CHES (pH 9 to 10) . The reaction mixture (100 µL) consisting of 50 mM buffers, 500 µM SAM and 100 µg/mL rOrf30 was incubated at 30 °C for 1 hr. (**C**) The reaction mixture (100 µL) consisting of 50 mM NaPB (pH 8), 500 µM SAM and 100 µg/mL rOrf30 was incubated for 1 hr at different temperatures to determine the optimum temperature for the rOrf30 enzyme reaction. (**D**) In the kinetic assays for rOrf30, the reaction mixture (100 µL) consisting of 50 mM NaPB (pH 8), 250 – 2,000 µM SAM and 100 µg/mL rOrf30 was incubated for 30 °C at 1 hr. The K_m and K_{cat} values were calculated from curve fitting to the Michaelis-Menten equation using software GraphPad Prism8. The kinetic analysis was performed in triplicates.

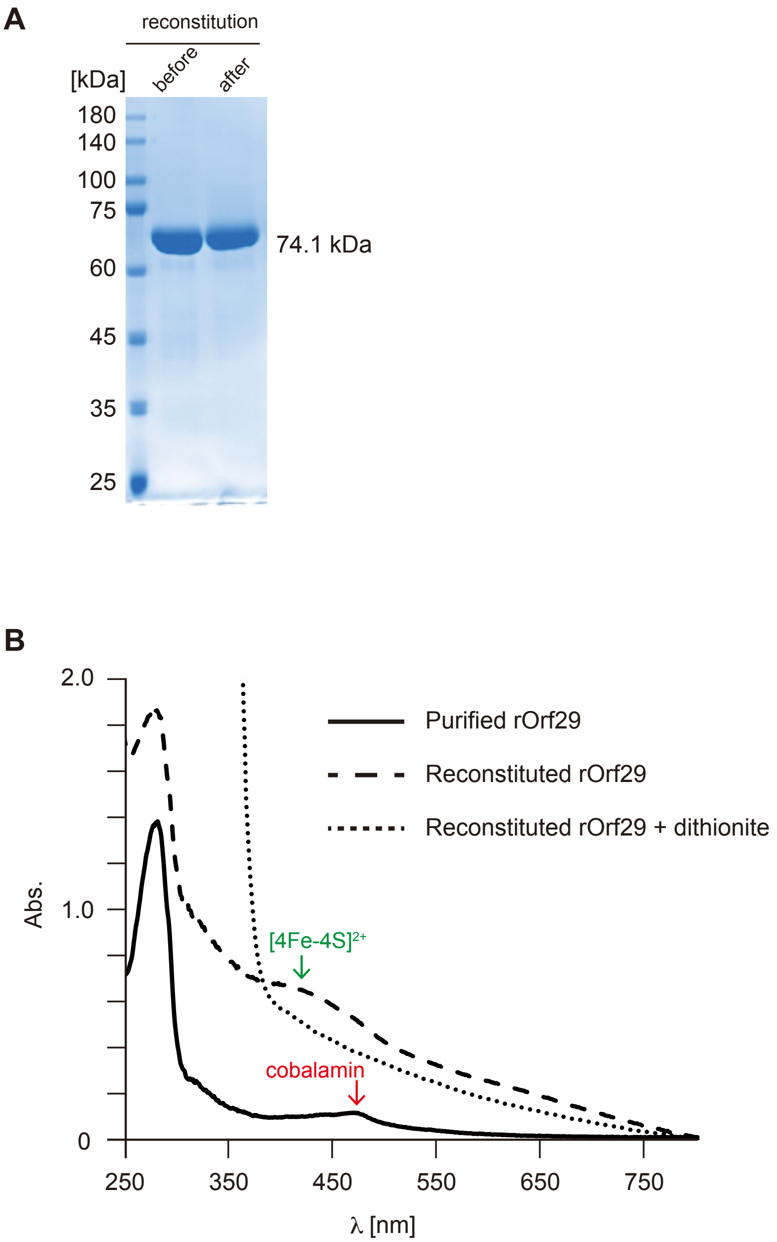

Figure S7. Overexpression of rOrf29 and reconstitution of the iron-sulfur cluster. (**A**) The purified rOrf29 was subjected to SDS-PAGE and was then stained with CBB R-250. (**B**) The purified rOrf29 solution was incubated in the presence of $FeSO_4(NH_4)_2SO_4$ $FeCl_3$, and Na_2S to reconstitute the iron-sulfur cluster. The presence of the oxidized $[4Fe\text{-}4S]^{2+}$ cluster was confirmed by UV-Vis spectroscopic analysis be observing the characteristic absorption at 420 nm. After treatment with sodium dithionite, the bleached absorption at 420 nm confirmed that the iron-sulfur cluster could be reduced to its active form $[4Fe\text{-}4S]^{+}$.

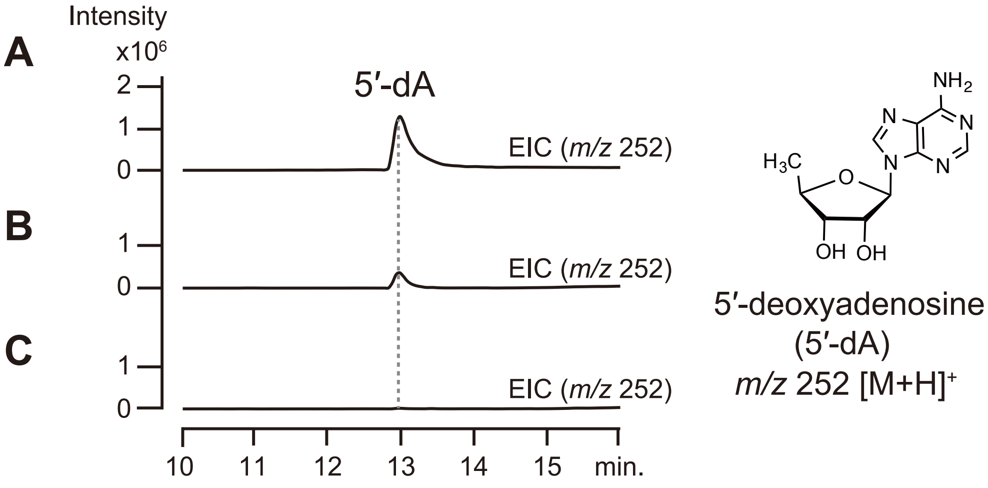

Figure S8. Detection of Ado-CH_3 in the rOrf29 reaction. The standard compound of Ado-CH_3 was analyzed by HPLC-ESI-MS (**A**), and the enzyme reaction with rOrf29 (**B**) or without rOrf29 (**C**) was analyzed by HPLC-ESI-MS. EICs for *m/z* 252 are shown.

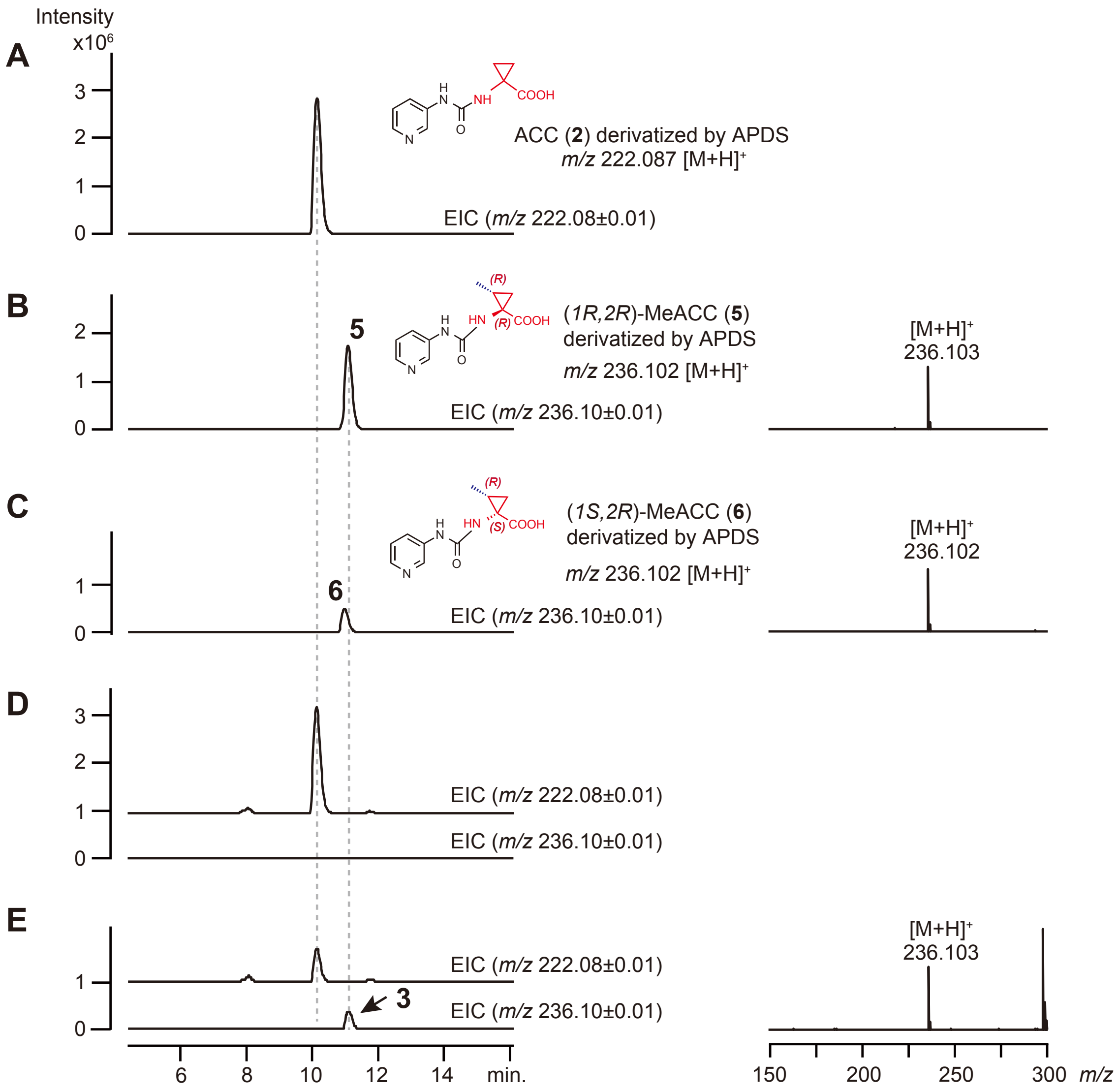

Figure S9. Enzymatic synthesis of MeACC (3) by a sequential reaction with rOrf29 and rOrf30. Standard compounds, ACC (**2**) (**A**), (*1R,2R*)-MeACC (**5**) (**B**), (*1S,2R*)-MeACC (**6**) (**C**) were derivatized by APDS and analyzed by HPLC-HR-ESI-MS. (**D**) Compound **2** was incubated with the reconstituted rOrf29. (**E**) Compound **1** was incubated with the reconstituted rOrf29, and then rOrf30 was added into the resulting enzyme reaction. These enzyme reaction mixtures were analyzed by HPLC-HR-ESI-MS. EICs for **2** (*m/z* 222.08±0.01) and **3** (*m/z* 236.10±0.01) are shown.

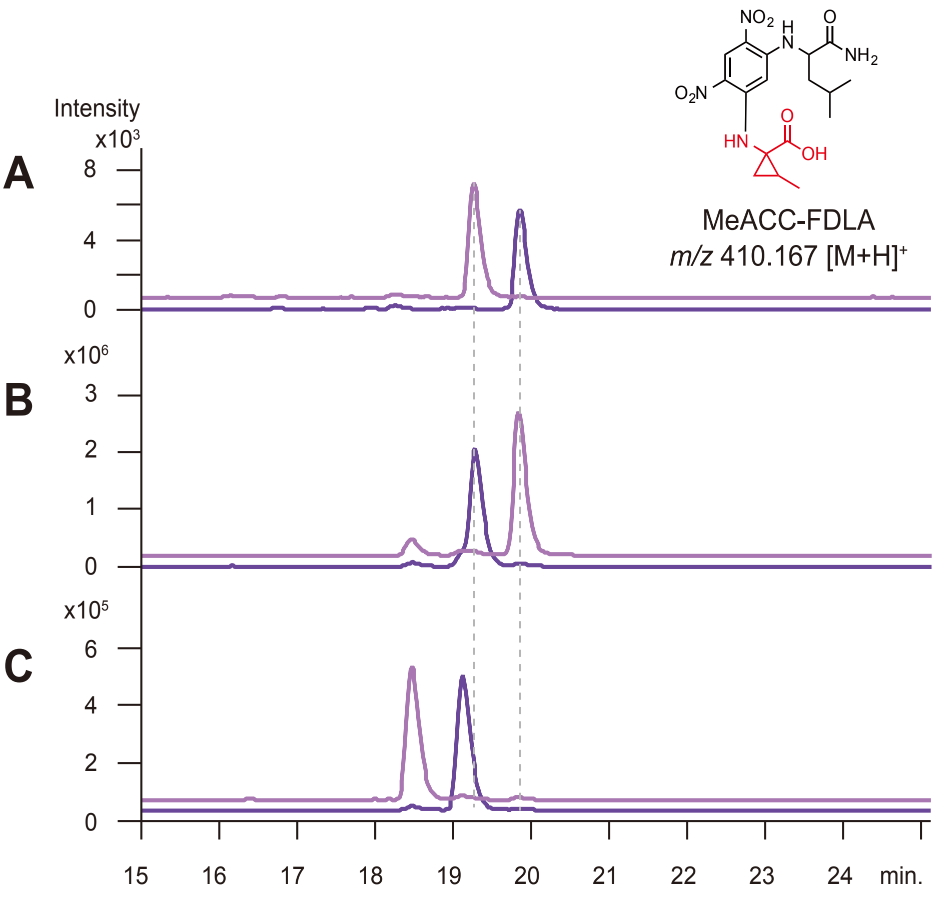

Figure S10. Determination of the absolute configuration of MeACC (3). The enzymatically synthesized **3** (**A**), and the chemically synthesized standards, (*1R,2R*)-MeACC (**5**) (**B**) and (*1S,2R*)-MeACC (**6**) (**C**), were derivatized using the Marfey's reagents, N^{α}-(5-Fluoro-2,4-dinitrophenyl)-L-leucinamide (L-FDLA) and N^{α}-(5-Fluoro-2,4-dinitrophenyl)-D-leucinamide (D-FDLA). The resulting derivatives were analyzed by HPLC-HR-ESI-MS. EICs for *m/z* 410.16±0.01 are shown, and the L-FDLA and D-FDLA derivatives are shown in light purple and purple, respectively.

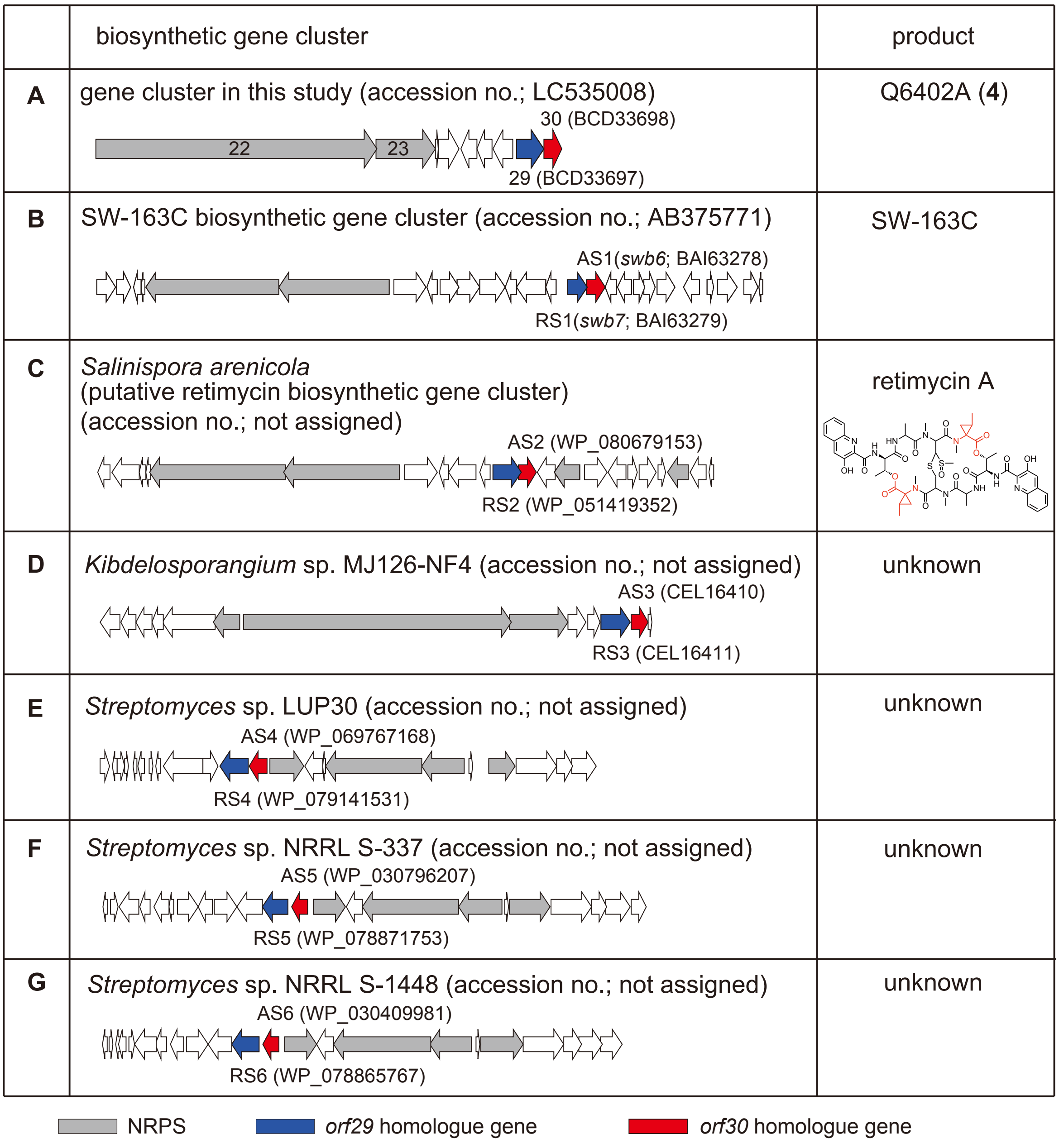

Figure S11. MeACC cluster homologues. (**A**) The MeACC cluster identified in this study. (**B**) SW-163C biosynthetic gene cluster. (**C**) Putative retimycin biosynthetic gene cluster. (**D**) The MeACC homologue cluster identified in *Kibdelosporangium* sp. MJ126-NF4. (**E**). The MeACC homologue cluster identified in *Streptomyces* sp. LUP30. (**F**) The MeACC homologue cluster identified in *Streptomyces* sp. NRRL S-337. (**G**) The MeACC homologue cluster identified in *Streptomyces* sp. NRRL S-1448.

2. Supplementary Tables

Table S1. Plasmids and strains used in this study

plasmid or strain	description	reference or source
**plasmids**		
pKU518	Amp^r, Neo^r (*aphII*), *ϕ*BT1-integrating BAC vector	(1)
pKU518_MeACC	pKU518 derivative carrying the MeACC cluster (59 kbp)	this study
pKU518_MeACC_*Δorf29*	pKU518 derivative carrying the MeACC cluster with the inactivated *orf29* gene	this study
pKU518_MeACC_*Δorf30*	pKU518 derivative carrying the MeACC cluster with the inactivated *orf30* gene	this study
pHSA81	expression vector for *Streptomyces* strain	(2)
pHSA81_*orf30*_C8His	pHSA81 derivative carrying the *orf30* gene for the overexpression	this study
pRKSUF017	pRKNMC derivative carrying the *suf* gene cluster, *sufABCDSE-ynhG*	(3)
pETDuet-1	co-expression vector for *E. coli*	Merck
pETDuet-1_*orf29_orf30*	pETDuet-1 derivative carrying the *orf29* and *orf30* genes	this study
pETDuet-1_*orf29*	pETDuet-1 derivative carrying the *orf29* gene	this study
pRSFDuet-1	co-expression vector for *E. coli*	Merck
pBAD24	expression vector for *E. coli*	(4)
pBAD24-BtuCEDFB	pBAD24 derivative carrying the cobalamin uptake gene cluster, *btuCEDFB*	this study
pET28	expression vector for *E. coli*	Merck
pET28_*orf29*	pET28 derivative carrying the *orf29* gene	this study
***Streptomyces* strains**		
Streptomyces violaceusniger 4521-SVS3	Streptomyces strain having a putative Q6402A biosynthetic gene cluster	this study
Streptomyces lividans TK23	heterologous host strain for gene expression experiments	(5)
TK23_MeACC	TK23 derivative harboring pKU518_MeACC	this study
TK23_empty	TK23 derivative harboring pKU518	this study
TK23_MeACC_Δ*orf29*	TK23 derivative harboring pKU518_MeACC_*Δorf29*	this study
TK23_MeACC_Δ*orf30*	TK23 derivative harboring pKU518_MeACC_*Δorf30*	this study
TK23_rOrf30/C8His	TK23 derivative harboring pHSA81_*orf30*_C8His	this study
***E. coli* strains**		
C41(DE3)	*E. coli* BL21(DE3) derivative for overexpression experiments; F$^-$ *ompT hsdS*$_B$(r$_B^-$ m$_B^-$) *gal dcm* (DE3)	Merck
EcSuf	*E. coli* C41(DE3) derivative harboring pRKSUF017	this study
EcSuf_empty	EcSuf derivative harboring pETDuet-1	this study
EcSuf_*orf29_orf30*	EcSuf derivative harboring pETDuet-1_*orf29_orf30*	this study
EcSuf_*orf29*	EcSuf derivative harboring pETDuet-1_*orf29*	this study
BL21(DE3)	fhuA2 [lon] ompT gal (λ DE3) [dcm] ΔhsdS λ DE3 = λ sBamHIo ΔEcoRI-B int::(lacI::PlacUV5::T7 gene1) i21 Δnin5	Merck
EcSufBtu	BL21(DE3) derivative harboring pRKSUF017 and pBAD24-BtuCEDFB	this study
EcSufBtu_*orf29*	EcSufBtu derivative harboring pET28_*orf29*	this study

References

(1) Komatsu M., Komatsu K., Koiwai H., Yamada Y., Kozone I., Izumikawa M., Hashimoto J., Takagi M., Omura S., Shin-ya K., Cane DE., Ikeda H., *ACS Synth. Biol.* **2013**, 2, 384–396.

(2) Matsumoto M., Hashimoto Y., Saitoh Y., Kumano T., Kobayashi M., *Biosci Biotechnol Biochem*. **2016**, Jun;80(6):1230-7

(3) Takahashi Y., Tokumoto U., *J Biol Chem*. **2002**, Aug 9;277(32):28380-3. Epub 2002 Jun 27.

(4) Guzman, L.M.; Belin, D.; Carson, M.J.; Beckwith, J. Tight regulation, modulation, and high-level expression by vectors containing the arabinose PBAD promoter. *J. Bacteriol*. **1995**, 177, 4121-4130

(5) Kieser T, Bibb MJ, Buttner MJ, Chater KF, Hopwood DA. **2000**, John Innes Foundation, Norwich, United Kingdom.

Table S2. Primers used in this study

oligonucleotides	sequences (5' → 3')	experiments
orf21-F	ATGACCGTCGCCGCATCAGTCAACGAGAACCTGGC	PCR screening for BAC clone
orf21-R	TCAGACGCCGGAGTACGAGTGCTTCCCTGAGACG	PCR screening for BAC clone
orf30-F	ATGCGCCTGGACACCTTCGATCTGGCCACGCTCTC	PCR screening for BAC clone
orf30-R	TCAGATCGGCCATGTGTCATGGTCCCCGCGGTGCG	PCR screening for BAC clone
pHSA81_*orf30*-F	GGAATTC**CATATG**CGCCTGGACACCTTCGATCTGGCC	overexpression of rOrf30
pHSA81_C8His-*orf30*-R	ACC**AAGCTT**TCAGTGGTGGTGGTGGTGGTGGTGGTGGATCG GCCATGTGTC	overexpression of rOrf30
pETDuet-1_*orf29*-F	GGAATTC**CATATG**CCTGGGAACATTCACATTCAG	co-expression of the *orf29* and *orf30* genes
pETDuet-1_*orf29*-R	**GATATC**TCAGCCGCCCACCCTCTCCTGTGC	co-expression of the *orf29* and *orf30* genes
pETDuet-1_*orf30*-F	CGC**GGATCC**GCGCCTGGACACCTTCGATCTGGCC	co-expression of the *orf29* and *orf30* genes
pETDuet-1_*orf30*-R	ACC**AAGCTT**TCAGATCGGCCATGTGTCATGGTC	co-expression of the *orf29* and *orf30* genes
pET28_*orf29*-F	GGAATTC**CATATG**CCTGGGAACATTCACATTCAG	overexpression of rOrf29
pET28_*orf29*-R	ACC**AAGCTT**TCAGCCGCCCACCCTCTCCTGTGC	overexpression of rOrf29

The restriction enzyme sites used for cloning are shown in bold.

Table S3. Sequences of the synthetic DNA fragments for construction of the plasmid pBAD24_BtuCEDFB

DNA fragment	genes	Sequence (5' → 3')
Fragment 1<br>(1215 bp)	*btuC*<br>*btuE'*	**CCATGG**TGCTGACACTTGCCCGCCAACAACAGCGACAAAATATTCGCTGGTTATTATGCCTGTCAGTTTTGATG<br>CTGCTGGCGCTTCTCTTAAGCCTTTGCGCCGGTGAACAATGGATCTCGCCAGGTGACTGGTTTACTCCTCGTG<br>GCGAACTGTTCGTCTGGCAAATTCGCCTGCCACGTACGCTGGCTGTATTGCTGGTTGGTGCGGCGCTGGCTAT<br>ATCCGGCGCTGTAATGCAGGCGTTGTTTGAAAATCCTCTGGCAGAACCTGGACTACTTGGCGTCTCTAACGGC<br>GCAGGCGTGGGGCTTATCGCCGCGGTATTGCTTGGGCAAGGGCAACTCCCCAACTGGGCGCTAGGGCTGTGT<br>GCGATTGCTGGCGCGCTTATCATCACTTTAATACTCTTACGTTTCGCCCGTCGTCATCTTTCGACCAGTCGGTTA<br>TTGCTGGCTGGCGTTGCATTAGGGATTATCTGTAGCGCACTAATGACGTGGGCTATCTACTTTTCCACCTCAGT<br>TGATTTGCGTCAGCTGATGTACTGGATGATGGGCGGTTTTGGCGGCGTAGACTGGCGGCAAAGCTGGCTGAT<br>GCTGGCATTGATCCCCGTGTTGTTGTGGATCTGTTGTCAGTCCAGGCCGATGAATATGTTAGCACTTGGCGAG<br>ATCTCGGCGCGGCAACTGGGTTTACCCCTGTGGTTCTGGCGCAATGTGCTGGTGGCAGCGACCGGCTGGATG<br>GTTGGCGTCAGTGTGGCGCTGGCGGGTGCTATCGGCTTTATTGGTCTGGTGATCCCCCATATTCTCCGGTTGT<br>GTGGTTTAACCGATCATCGCGTATTACTTCCCGGCTGCGCGCTGGCAGGGGCGAGCGCATTGCTGCTGGCCG<br>ATATTGTAGCGCGCCTGGCATTAGCTGCCGCAGAGCTGCCTATTGGCGTGGTCACCGCAACGTTAGGTGCGC<br>CGGTGTTTATCTGGTTATTGTTAAAAGCAGGACGTTAGCCGCAAAAAGACGGTCTATGATTAAAAGCTAAGATTT<br>TTACTGACCACTAAGGAGGTAAACGATGCAAGATTCCATTCTGACGACCGTAGTGAAAGATATCGACGGTGAA<br>GTGACCACGCTGGAGAAGTTCGCCGGTAATGTGCTGTTGATTGTCAATGTCGCCTCAAAGTGTGGCTTAACGC<br>CGCAATATGAGCAGTTGGAGAATATTCAGAAAGCCTGGGT**CGATCG**
Fragment 2<br>(1961 bp)	*btuE''*<br>*btuD*<br>*btuF*<br>*btuB'*	**CGATCG**AGGTTTTATGGTGCTGGGATTCCCGTGCAACCAGTTTCTGGAACAAGAACCGGGCAGCGATGAAGAG<br>ATTAAAACTTACTGTACCACCACATGGGGGGTGACGTTCCCGATGTTCAGTAAGATTGAAGTTAATGGCGAAGG<br>ACGCCATCCGCTGTATCAAAAATTGATTGCCGCAGCGCCGACCGCAGTCGCGCCGGAAGAGAGCGGATTCTA<br>TGCCCGTATGGTCAGCAAAGGCCGTGCACCGCTGTACCCGGATGATATTTTATGGAATTTTGAAAAATTCCTGG<br>TTGGCAGGGACGGAAAAGTCATCCAGCGTTTTTCCCCGGATATGACGCCGGAAGATCCCATTGTGATGGAAAG<br>CATTAAACTGGCGTTGGCAAAATAATGTCTATTGTGATGCAGTTACAAGATGTTGCGGAATCTACCCGCCTGGG<br>GCCGCTTTCTGGCGAGGTTCGGGCTGGGGAGATCCTGCACCTGGTGGGGCCGAATGGCGCGGGTAAGAGTA<br>CCTTACTGGCGCGAATGGCCGGAATGACCAGCGGTAAGGGAAGCATTCAGTTCGCGGGGCAACCACTGGAAG<br>CATGGTCCGCAACAAAACTCGCGCTGCATCGCGCCTATCTTTCACAACAGCAGACGCCGCCGTTTGCAACGCC<br>GGTCTGGCACTACCTGACACTGCATCAGCACGATAAAACGCGTACCGAACTACTGAATGATGTCGCAGGGGCG<br>CTGGCTCTTGATGACAAACTCGGACGTAGCACCAATCAACTTTCCGGCGGTGAATGGCAACGCGTACGTCTTG<br>CTGCGGTGGTGTTGCAAATCACACCACAAGCCAATCCCGCAGGCCAATTGCTGCTTCTTGATGAGCCGATGAA<br>CAGTCTTGATGTTGCGCAACAAAGTGCGTTAGACAAAATTCTGAGCGCGCTGTGTCAGCAAGGACTGGCGATT<br>GTGATGAGCAGTCACGATCTCAACCACACATTGCGTCATGCGCATCGGGCGTGGTTGCTAAAAGGTGGAAAAA<br>TGCTGGCCAGTGGACGCAGGGAAGAGGTGCTCACGCCGCCAAATCTGGCGCAGGCCTATGGGATGAATTTTC<br>GCCGTCTGGATATCGAAGGTCACAGAATGCTGATTTCGACCATCTGATGGCTAAGTCACTGTTCAGGGCGCTG<br>GTCGCCCTGTCTTTTCTTGCGCCACTGTGGCTCAACGCCGCGCCGCGCGTCATCACGCTTTCTCCCGCCAACA<br>CTGAACTTGCCTTTGCCGCCGGGATCACGCCGGTTGGGGTCAGCAGCTATTCCGACTATCCTCCACAAGCGCA<br>AAAGATTGAGCAGGTTTCCACCTGGCAGGGGATGAATCTGGAACGCATTGTCGCGCTGAAACCCGATCTGGTG<br>ATTGCCTGGCGTGGAGGTAATGCCGAGCGGCAGGTTGACCAGCTGGCTTCGCTGGGAATAAAAGTGATGTGG<br>GTCGATGCGACAAGCATTGAACAAATTGCCAATGCGTTACGTCAACTGGCCCCCTGGAGTCCGCAACCAGACA<br>AGGCCGAACAAGCCGCGCAATCCCTGCTGGATCAGTACGCGCAATTGAAAGCGCAATATGCTGATAAACCTAA<br>AAAACGTGTTTTTCTGCAATTCGGCATTAATCCGCCATTTACCAGTGGAAAAGAGTCGATTCAGAACCAGGTAC<br>TCGAAGTTTGTGGCGGAGAAAACATCTTTAAAGACAGCCGGGTTCCCTGGCCGCAAGTTAGCCGCGAACAGGT<br>GTTAGCACGCTCGCCACAGGCGATTGTCATTACAGGCGGACCGGACCAAATTCCTAAAATCAAACAATACTGG<br>GGTGAACAGCTCAAAATTCCCGTTATTCCTCTCACGAGTGACTGGTTTGAACGTGCAAGCCCACGTATTATCCT<br>CGCTGCACAACAGCTCTGTAATGCGCTTTCACAGGTAGATTGATGATTAAAA**AAGCTTGGTACC**
Fragment 3<br>(1847 bp)	*btuB''*	**AAGCTT**CGCTGCTGACGGCGTGTTCCGTCACGGCATTTTCCGCTTGGGCACAGGATACCAGCCCGGATACTCT<br>CGTCGTTACTGCTAACCGTTTTGAACAGCCGCGCAGCACTGTGCTTGCACCAACCACCGTTGTGACCCGTCAG<br>GATATCGACCGCTGGCAGTCGACCTCGGTCAATGATGTGCTGCGCCGTCTTCCGGGCGTCGATATCACCCAAA<br>ACGGCGGTTCAGGTCAGCTCTCATCTATTTTTATTCGCGGTACAAATGCCAGTCATGTGTTGGTGTTAATTGATG<br>GCGTACGCCTGAATCTGGCGGGGGTGAGTGGTTCTGCCGACCTTAGCCAGTTCCCTATTGCGCTTGTCCAGC<br>GTGTTGAATATATCCGTGGGCCGCGCTCCGCTGTTTATGGTTCCGATGCAATAGGCGGGGTGGTGAATATCAT<br>CACGACGCGCGATGAACCCGGAACGGAAATTTCAGCAGGGTGGGGAAGCAATAGTTATCAGAACTATGATGTC<br>TCTACGCAGCAACAACTGGGGGATAAGACACGGGTAACGCTGTTGGGCGATTATGCCCATACTCATGGTTATG<br>ATGTTGTTGCCTATGGTAATACCGGAACGCAAGCGCAGACAGATAACGATGGTTTTTTAAGTAAAACGCTTTAT<br>GGCGCGCTGGAGCATAACTTTACTGATGCCTGGAGCGGCTTTGTGCGCGGCTATGGCTATGATAACCGTACCA<br>ATTATGACGCGTATTATTCTCCCGGTTCACCGTTGCTCGATACCCGTAAACTCTATAGCCAAAGTTGGGACGCC<br>GGGCTGCGCTATAACGGCGAACTGATTAAATCACAACTCATTACCAGCTATAGCCATAGCAAAGATTACAACTA<br>CGATCCCCATTATGGTCGTTATGATTCGTCGGCGACGCTCGATGAGATGAAGCAATACACCGTCCAGTGGGCA<br>AACAATGTCATCGTTGGTCACGGTAGTATTGGTGCGGGTGTCGACTGGCAGAAACAGACTACGACGCCGGGTA<br>CAGGTTATGTTGAGGATGGATATGATCAACGTAATACCGGCATCTATCTGACCGGGCTGCAACAAGTCGGCGA<br>TTTTACCTTTGAAGGCGCAGCACGCAGTGACGATAACTCACAGTTTGGTCGTCATGGAACCTGGCAAACCAGC<br>GCCGGTTGGGAATTCATCGAAGGTTATCGCTTCATTGCTTCCTACGGGACATCTTATAAGGCACCAAATCTGGG<br>GCAACTGTATGGCTTCTACGGAAATCCGAATCTGGACCCGGAGAAAAGCAAACAGTGGGAAGGCGCGTTTGAA<br>GGCTTAACCGCTGGGGTGAACTGGCGTATTTCCGGATATCGTAACGATGTCAGTGACTTGATCGATTATGATGA<br>TCACACCCTGAAATATTACAACGAAGGGAAAGCGCGGATTAAGGGCGTCGAGGCGACCGCCAATTTTGATACC<br>GGACCACTGACGCATACTGTGAGTTATGATTATGTCGATGCGCGCAATGCGATTACCGACACGCCGTTGTTAC<br>GCCGTGCTAAACAGCAGGTGAAATACCAGCTCGACTGGCAGTTGTATGACTTCGACTGGGGTATTACTTATCA<br>GTATTTAGGCACTCGCTATGATAAGGATTACTCATCTTATCCTTATCAAACCGTTAAAATGGGCGGTGTGAGCTT<br>GTGGGATCTTGCGGTTGCGTATCCGGTCACCTCTCACCTGACAGTTCGTGGTAAAATAGCCAACCTGTTCGAC<br>AAAGATTATGAGACAGTCTATGGCTACCAAACTGCAGGACGGGAATACACCTTGTCTGGCAGCTACACCTTCTA<br>A**TCTAGACTCGAG**

The restriction enzyme sites used for cloning are shown in bold.

Table S4. Orf29 homologs used in the phylogenetic analysis

ID No.	organisms	putative functions	amino acid length	Identity with Orf29 (%)	accession No.
Orf29	*Streptomyces violaceusniger* 4521-SVS3	B12-binding domain-containing radical SAM protein	640	100	BCD33697
RS1 (Swb7)	*Streptomyces sp.* SNA15896	hypothetical protein	478	69	BAI63279
RS2	*Salinispora arenicola*	radical SAM protein	612	69	WP_051419352
RS3	*Kibdelosporangium sp.* MJ126-NF4	Radical SAM domain protein	644	71	CEL16411
RS4	*Streptomyces sp.* LUP30	radical SAM protein	642	64	WP_079141531
RS5	*Streptomyces sp.* NRRL S-337	radical SAM protein	640	66	WP_078871753
RS6	*Streptomyces sp.* NRRL S-1448	radical SAM protein	640	66	WP_078865767
RS7	*Streptomyces sp.* ADI95-16	radical SAM protein	621	67	WP_123082798
RS8	*Streptomyces sp.* WM6372	B12-binding domain-containing radical SAM protein	621	66	WP_053690759
RS9	*Streptomyces sp.* 3211.1	radical SAM superfamily enzyme YgiQ	655	65	RIA73630
RS10	*Streptomyces lydicus*	radical SAM protein	639	68	WP_078616973
RS11	*Streptomyces sp.* MOE7	B12-binding domain-containing radical SAM protein	627	68	ARH95039
RS12	*Streptomyces sp.* 76	radical SAM superfamily enzyme YgiQ	644	68	PIF72286
RS13	*Streptomyces sp.* 76	radical SAM protein	639	68	WP_099873486
RS14	*Streptomyces alboniger*	radical SAM protein	642	65	WP_150477685
RS15	*Streptomyces caeruleatus*	radical SAM protein	642	64	WP_079056031
RS16	*Streptomyces hyalinus*	hypothetical protein EHYA_08028	684	64	GCE00303
RS17	*Actinokineospora enzanensis*	radical SAM protein	639	67	WP_156892875
RS18	*Streptomyces sp.* NRRL S-920	radical SAM protein	642	64	WP_078594250
RS19	*Streptomyces caeruleatus*	radical SAM protein	610	65	KUO03048
RS20	*Streptomyces scabrisporus*	B12-binding domain-containing radical SAM protein	660	65	OPC79404
RS21	*Streptomyces sp.* 61	radical SAM superfamily enzyme YgiQ	658	64	PIG45441
RS22	*Saccharomonospora halophila*	B12-binding domain-containing radical SAM protein	665	63	WP_019812377
RS23	*Micrococcales bacterium*	B12-binding domain-containing radical SAM protein	580	65	PID55382
RS24	*Streptomyces sp.* NEAU-D10	radical SAM protein	633	53	WP_128510192
RS25	*Streptomyces sp.* ADI95-16	Radical SAM superfamily protein	655	66	AYV31161
RS26	*Streptomyces sp.* CB02058	hypothetical protein AMK10_20405	613	55	OKI94760
RS27	*Streptomyces sp.* NRRL S-337	radical SAM protein	631	52	WP_051798488
RS28	*Streptomyces sp.* NRRL S-1448	radical SAM protein	648	52	WP_078865991
RS29	*Streptomyces sp.* 76	radical SAM family protein	656	50	PIF88042
RS30	*Streptomyces lydicus*	radical SAM family protein	630	52	WP_079271693
RS31	*Streptomyces sp.* MOE7	hypothetical protein	620	52	ARH95601
RS32	*Streptomyces ipomoeae* 91-03	radical SAM domain protein	595	53	EKX67605
RS33	*Streptomyces sp.* SDr-06	radical SAM protein	622	45	WP_114040836
RS34	*Streptomyces sp.* PsTaAH-130	radical SAM protein	621	45	WP_111587184
RS35	*Streptomyces*	radical SAM protein	621	45	WP_079172442

RS36	*Streptomyces sp.* S816	radical SAM protein	621	45	WP_136103222
RS37	*Pseudomonas cepacia*	Hopanetetrol cyclitol ether synthase (HpnJ)	473	no identity	U1XSN3
RS38	*Rhodobacter capsulatus*	Mg-protoporphylin IX monomethyl ester anaerobic oxidative cyclase (BchE)	575	20	P26168
RS39	*Bacillus megaterium*	Radical SAM protein (OxsB)	744	31	O24770
RS40	*Chlorobaculum tepidum*	Bacteriochlorophyll C12 methyltransferase (BchR)	456	21	Q8KCU0
RS41	*Chlorobaculum tepidum*	Bacteriochlorophyll C8 methyltransferase (BchQ)	451	22	Q8KBK9
RS42	*Streptomyces roseochromogenus*	Cyclobiocin pyrrole-2-2carboxy methyltransferase (CloN6)	561	35	Q8GHB6
RS43	*Chondromyces crocatus*	Chondrochloren A methyltransferase (CndI)	700	25	B9ZUJ4
RS44	*Streptomyces wedmorensis*	2-hydroxyethyl phosphanate methyltransferase (Fom3)	534	23	Q56184
RS45	*Micromonospora olivasterospora*	Fortimicin KL1 methyltransferase (Fms7)	553	26	Q50258
RS46	*Micromonospora echinospora*	Gentamicin N-methyltransferase (GntE)	634	24	Q6QVU0
RS47	*Micromonospora echinospora*	Gentamicin C-6' methyltransferase (GenK)	638	24	Q70KE5
RS48	*Rhodopseudomonas palustris*	Hopanoid C2-methyltransferase (HpnP)	527	24	B3QHD1
RS49	*Methylococcus capsulatus*	Hopanoid C3-methyltransferase (HpnR)	515	20	Q60AV6
RS50	*Magnaporthe oryzae*	P-methyltransferase (PhpK)	399	no identity	Q51W50
RS51	*Streptomyces pactum*	Pactamycin C-methyltransferase (PctJ)	661	26	A8R0J3
RS52	*Streptomyces pactum*	Pactamycin C-methyltransferase (PctN)	575	no identity	A8R0J7
RS53	*Streptomyces pactum*	Pactamycin C-methyltransferase (PctO)	578	21	A8R0J8
RS54	*Streptomyces sp. SNA15896*	Thioacetal methyltransferase (Swb9)	679	29	D2KTX8
RS55	*Streptomyces cattleya*	Carbapenem intermediate methyltransferase (ThnK)	681	26	F8JND9
RS56	*Streptomyces laurentii*	Tryptophan 2-C-methhyltransferase (TsrM)	599	23	C0JRZ9

Table S5. Orf30 homologs used in the phylogenetic analysis

ID No.	organisms	putative functions	amino acid length	Identity with Orf30 (%)	accession No.
**bacteria**					
Orf30	*Streptomyces violaceusniger* 4521-SVS3	aminotransferase class I/II-fold PLP-dependent enzyme	402	100	BCD33698
GnmY	*Streptomyces sp.* CB01883	ACC synthase	433	40	ATY69574
AS1 (Swb6)	*Streptomyces sp.* SNA15896	aminotransferase	389	69	BAI63278
AS2	*Salinispora arenicola*	aminotransferase class I/II-fold PLP-dependent enzyme	405	66	WP_080679153
AS3	*Kibdelosporangium sp.* MJ126-NF4	PLP-dependent enzyme	382	72	CEL16410
AS4	*Streptomyces sp.* LUP30	aminotransferase class I/II-fold PLP-dependent enzyme	399	63	WP_069767168
AS5	*Streptomyces sp.* NRRL S-337	aminotransferase class I/II-fold PLP-dependent enzyme	398	62	WP_030796207
AS6	*Streptomyces sp.* NRRL S-1448	aminotransferase class I/II-fold PLP-dependent enzyme	398	62	WP_030409981
AS7	*Streptomyces sp.* 1222.5	cystathione beta-lyase	433	41	SEB65447
AS8	*Streptomyces sp.* 2231.1	aminotransferase class I/II-fold PLP-dependent enzyme	433	41	WP_093698510
AS9	*Streptomyces sp.* LUP30	aminotransferase class I/II-fold PLP-dependent enzyme	435	41	WP_069773618
AS10	*Streptomyces sp.* SPB074	aminotransferase class I/II-fold PLP-dependent enzyme	433	41	WP_050776683
AS11	*Streptomyces sp.* NRRL F-5630	aminotransferase class I/II-fold PLP-dependent enzyme	434	40	WP_051703302
AS12	*Streptomyces sp.* CB01201	hypothetical protein	446	39	PJN02064
AS13	*Penicillium citrinum*	ACC synthase	431	24	BAA92149
**plant**					
AS14	*Actinidia deliciosa*	ACC synthase	467	22	BAA31137
AS15	*Antirrhinum majus*	ACC synthase 3	434	24	AAC70353
AS16	*Arabidopsis thaliana*	ACC synthase 5	470	24	Q37001
AS17	*Arabidopsis thaliana*	ACC synthase 2	496	23	Q06402
AS18	*Brassica juncea*	ACC synthase	497	29	CAA51227
AS19	*Brassica oleracea*	ACC synthase	491	25	CAA57724
AS20	*Carica papaya*	ACC synthase	488	24	CAA72191
AS21	*Citrullus colocynthis*	ACC synthase	486	26	ABO76785
AS22	*Cucumis melo*	ACC synthase	481	23	ACO83163
AS23	*Cucumis melo*	ACC synthase	490	27	BAB18464
AS24	*Cucumis melo*	ACC synthase	445	23	ACG70850
AS25	*Cucumis sativus*	ACC synthase	481	23	ABI33818
AS26	*Cucumis sativus*	ACC synthase	445	24	ACT78959
AS27	*Cucurbita maxima*	ACC synthase	475	22	BAB47123
AS28	*Cucurbita maxima*	ACC synthase	485	25	BAB47124
AS29	*Cucurbita pepo*	ACC synthase	445	24	AHE81230
AS30	*Dendrobium hybrid cultivar*	ACC synthase	435	23	ABO68836
AS31	*Dendrobium hybrid cultivar*	ACC synthase	435	24	ABO68835
AS32	*Dendrobium hybrid cultivar*	ACC synthase	435	24	ACY72181
AS33	*Diospyros kaki*	ACC synthase	486	24	BAB89350
AS34	*Diospyros kaki*	ACC synthase	471	24	BAB89348
AS35	*Glycine max*	ACC synthase	484	24	ABB70230
AS36	*Gossypium hirsutum*	ACC synthase	475	24	ABJ99502
AS37	*Ipomoea nil*	ACC synthase	472	23	ABL67952

AS38	*Lupinus albus*	ACC synthase 4	446	23	AAF22108
AS39	*Lupinus albus*	ACC synthase 5	448	23	AAF22112
AS40	*Malus domestica*	ACC synthase	473	23	P37821
AS41	*Malus domestica*	ACC synthase 3b	445	25	BAE94691
AS42	*Malus sylvestris*	ACC synthase	454	24	1808332A
AS43	*Musa acuminata*	ACC synthase	463	26	AAQ13435
AS44	*Musa acuminata*	ACC synthase	439	26	CAA11158
AS45	*Oryza sativa indica*	ACC synthase	461	29	CAA65776
AS46	*Oryza sativa indica*	ACC synthase	487	23	AAA33888
AS47	*Paeonia lactiflora*	ACC synthase	492	23	AFT92042
AS48	*Pelargonium x hortorum*	ACC synthase	482	25	ABQ51839
AS49	*Pelargonium x hortorum*	ACC synthase	435	25	ABQ51842
AS50	*Picea glauca*	ACC synthase	447	24	ABM60747
AS51	*Pisum sativum*	ACC synthase	480	22	AAD04198
AS52	*Pisum sativum*	ACC synthase	487	25	AAD04199
AS53	*Prunus salicina*	ACC synthase 5	483	25	ABW03086
AS54	*Prunus salicina*	ACC synthase 1	470	24	ABW03081
AS55	*Pseudotsuga menziesii*	ACC synthase	460	22	ABM60752
AS56	*Pyrus pyrifolia*	ACC synthase	473	23	AGG19162
AS57	*Pyrus pyrifolia*	ACC synthase	446	25	BAA76388
AS58	*Rosa hybrid cultivar*	ACC synthase	480	24	AAQ88100
AS59	*Solanum lycopersicum*	ACC synthase 8	471	25	AF179247_1
AS60	*Solanum lycopersicum*	ACC synthase 5	471	23	AF179246_1
AS61	*Solanum lycopersicum*	ACC synthase	476	24	CAH56693
AS62	*Solanum tuberosum*	ACC synthase	441	23	CAA81749
AS63	*Solanum tuberosum*	ACC synthase	465	23	CAA81747
AS64	*Vigna radiata*	ACC synthase	472	25	AAA78273
AS65	*Vigna radiata var. radiata*	ACC synthase	368	26	Q01912

Table S6. Substrate specificity analysis of six A domains in the MeACC cluster.

Orf	Specificity-conferring code [a]										Predicted substrate [b]	Stachelhaus code match	Actual substrate
	235	236	239	278	299	301	322	330	331	517			
22_A1	D	F	W	N	I	G	M	V	H	K	Thr	100 %	Thr
22_A2	D	A	W	T	V	A	A	V	C	K	Phe	100 %	Phe
22_A3	D	M	T	K	L	A	V	V	S	K	Asn	60 %	Glu
22_A4	D	A	C	M	I	G	A	V	C	K	Phe	70 %	hydroxy-Trp
22_A5	D	I	Y	H	L	G	L	L	C	K	Hpg	90 %	Hpg
23_A	D	M	Y	S	Q	A	I	V	Y	K	Val	60 %	MeACC

[a] Residues defined according to Stachelhaus *et al.* and Challis *et al.*; residue numbering corresponds to gramicidin S synthetase A domain numbering (GrsA, PDB-ID : 1AMU).

[b] Retrieved from reports of the applied antiSMASH 5.0 gene cluster analysis, including substrate predictions based on the NRPSPredictor2 method.
